# Comparing the Properties of Bio-Polyols Based on White Mustard (*Sinapis alba*) Oil Containing Boron and Sulfur Atoms Obtained by Various Methods and Checking Their Influence on the Flammability of Rigid Polyurethane/Polyisocyanurate Foams

**DOI:** 10.3390/ma16093401

**Published:** 2023-04-26

**Authors:** Marcin Borowicz, Marek Isbrandt, Joanna Paciorek-Sadowska, Paweł Sander

**Affiliations:** Department of Chemistry and Technology of Polyurethanes, Faculty of Materials Engineering, Kazimierz Wielki University, J. K. Chodkiewicza Street 30, 85-064 Bydgoszcz, Poland

**Keywords:** mustard oil, bio-polyol, synthesis, boron, sulfur, bio-polyol properties, polyurethane foams, flammability tests

## Abstract

The article compares the properties of bio-polyols obtained from white mustard (*Sinapis alba*) seed oil, which contain boron and sulfur atoms. Each of the bio-polyols was prepared by a different method of testing the efficiency of the incorporation of boron and sulfur atoms. All synthesis methods were based on the epoxidation of unsaturated bonds followed by the opening of epoxy rings by compounds containing heteroatoms. Two of the bio-polyols were subjected to additional esterification reactions of hydroxyl groups with boric acid or its ester. Three new bio-polyols were obtained as a result of the performed syntheses. The synthesized compounds were subjected to detailed physicochemical (physical state, color, smell, density, viscosity and pH), analytical (hydroxyl number, acid number, water content, content of C, H, N, S, O, B elements and GPC analysis), spectroscopic (FTIR, ^1^H NMR and ^13^C NMR) and thermal (DSC) tests. The obtained results allowed for a detailed characterization of the synthesized bio-polyol raw materials. Their suitability for obtaining polyurethane materials was also determined. The synthesized compounds have been found to be an interesting alternative to petrochemical polyols. The influence of the synthesized compounds on the flammability of polyurethane materials was tested experimentally. On the basis of this testing, a number of rigid polyurethane/polyisocyanurate foams were obtained, which were then subjected to flammability tests with the methods of horizontal and vertical burning, limiting oxygen index (LOI) and using the cone calorimeter. Based on this research, it was found that the presence of sulfur and boron heteroatoms reduced the flammability of polyurethane materials based on synthesized bio-polyols.

## 1. Introduction

The global production of polyurethane plastics in 2021 amounted to 21.5 million tons, which accounted for 5.5% of the production of all polymer plastics. This shows how large the polyurethane industry is on a global scale [1]. In 2021, the size of the global bio-polyol market was estimated at USD 4.4 billion. It is expected that by 2027 this market will reach USD 6.9 billion, achieving a compound annual growth rate of 9.5%. This indicates a dynamic growth of this market, which will be caused by stringent legal regulations that will strive to limit the use of petroleum-based polyols in favor of raw materials from renewable sources [2]. The further scientific and technical development of civilization causes a global crisis. The reason for this crisis is the disturbance of relations between humanity and the natural environment. Consequently, this leads to the destruction of nature. Balancing the development of civilization and the proper functioning of nature is extremely difficult and complicated. The doctrine that is supposed to connect these two spheres is sustainable development. It presents solutions aimed at protecting society (culture), the natural environment and the economy. The doctrine of sustainable development combines the natural environment and socio-economic development. It assumes the improvement of the quality of life of contemporary and future generations without disturbing the state of the natural environment [3,4,5]. Based on the assumptions of “green chemistry” and sustainable development, the concept of this article was created. It combines the principles of caring for the natural environment by obtaining bio-polyols based on plant raw materials, which can be an alternative to petrochemical polyols used in the production of polyurethane materials. In addition, the sulfur and boron atoms contained in the bio-polyols may reduce the flammability of polyurethane materials. Consequently, this may contribute to the improvement of people’s safety; for example, during a fire [6].

Many research centers around the world are conducting research aimed at the use of vegetable oils (VO) for the synthesis of polyurethane (PU) materials. Scientists chose this direction of research due to three factors. The first is protecting the natural environment by replacing petrochemical polyols from the processing of crude oil with bio-polyols of plant origin. Second, vegetable oils are easily renewable, unlike fossil feedstock. Third, principles of sustainable development and green chemistry are applied in the chemical industry [7,8,9]. A separate issue is the development of the PU industry toward the use of renewable raw materials, including vegetable oils. The bio-polyol industry is developing very dynamically. In 2015, the world production of these raw materials amounted to 1.9 million tons. It is estimated that production will increase year by year by about 1% until 2024 [6,10,11]. This development is also reflected in scientific research. There are many articles on the synthesis of bio-polyols based on such oils as rapeseed [12], palm [13], sunflower [14], corn [15], soybean [16], linseed [17,18] and castor [19], evening primrose [20] and mustard [6] oils. The reasons for their use are mainly their environmentally friendly nature, easy renewal and as an alternative to petrochemical raw materials.

Currently, bio-polyols produced from VO are a rapidly growing base of raw materials used for the synthesis of PU foams. In Europe, rapeseed oil [7] and sunflower oil [21] are mainly used to produce bio-polyols from readily renewable raw materials. In turn, in Asia, palm and coconut oil are mainly used for this purpose [22]. On the other hand, in North America, mainly soybean oil is used [23]. The implementation of vegetable oils in the polyurethane industry as an alternative to using fossil raw materials is primarily for ecological reasons, as well as the increasing prices of crude oil and gas [24].

The production of mustard seeds in 2021 reached 532.8 thousand tons [25]. This shows the potential for oil production. Mustard oil (MO) is a light-yellow liquid with a characteristic mustard smell. The main components of MO are glycerides of the following acids: erucic, linoleic, oleic and icosenic. The percentage of unsaturated fatty acids in this oil is over 96% of all fatty acids. Therefore, MO is classified as the most unsaturated VO available on the market. Due to the high degree of unsaturation, it is possible to modify MO to obtain bio-polyols with a hydroxyl value from several dozen to over several hundred mg KOH/g [26].

Research in the field of polyurethane foams mainly concerns the improvement of their functional properties and, in particular, focuses on reducing their flammability. The current trend is to conduct research aimed at developing reactive polyols containing heteroatoms of elements that reduce the flammability of polyurethane material based on them [27]. Paciorek-Sadowska et al. used polyols based on boric acid derivatives and diols (e.g., 1,3-propanediol, 1,4-butanediol). These compounds containing boron heteroatoms as modified polyols were characterized by a decrease in the flammability of polyurethane/polyisocyanurate foams based on them [28]. Rao et al. synthesized a polyester polyol containing phosphorus heteroatoms from dimethyl methylphosphonate and diethanol amine. The synthesized polyol was used in the formulation to obtain flexible polyurethane foams, which were characterized by lower flammability in comparison with the used reference foam [29]. Bhakri et al. synthesized a polyurethane resin by the non-isocyanate method. They used 3-aminopropyltriethoxysylane to introduce a silicon heteroatom into the molecule [30]. Research on the preparation of polyol raw materials containing heteroatoms has been conducted for many years. However, the introduction of heteroatoms classified as flame retardants into oleochemical raw materials used for the synthesis of bio-polyols is a scientific novelty.

Currently, there is an effort to eliminate the toxic halogen flame retardants of polyurethane materials. The use of halogen retardants has significant disadvantages from a toxicological and ecological point of view. This is associated with the risk of the emission of large amounts of toxic compounds during a fire. During the thermal decomposition of the halogen-containing material, gases such as hydrogen chloride are released. This gas is highly irritating and toxic to humans [31]. The introduction of heteroatoms belonging to the group of flame retardants into the structures of polyol raw materials is an interesting alternative that may become popular in the polyurethane industry. In recent years, there have been many different scientific studies on the introduction of various elements from this group. The most common chemically incorporated flame-retardant elements include boron, sulfur, phosphorus, nitrogen, silicon, etc. [27,32,33,34,35]. The system of heteroatoms presented in this article (boron and sulfur) incorporated into the structure of bio-polyols based on white mustard oil is one of the very effective combustion inhibitors. The mechanism of their action is similar to that of halogen flame retardants and is at the same time devoid of their toxicity [27].

This article is a development of the technology of synthesis of bio-polyols based on mustard oil, which was presented in the authors’ earlier publications [6,33]. The main aim of the research was a comparison of the properties of bio-polyols based on MO containing boron and sulfur heteroatoms in their structure. Bio-polyols were prepared in several steps. They included, among others, the epoxidation of unsaturated bonds contained in the oil, the synthesis of ring-opening agents containing boron and sulfur heteroatoms, the opening of epoxy rings with suitable agents and/or the esterification of hydroxyl groups contained in the intermediate products. The obtained bio-polyols were subjected to basic physicochemical, analytical and spectroscopic tests in order to determine the possibility of using them as potential raw materials for the synthesis of PU polyurethane materials. The second aim of the research was to use the synthesized bio-polyols to obtain rigid polyurethane/polyisocyanurate foams. Then, they were subjected to flammability tests using four different methods in order to determine the influence of sulfur and boron atoms on the flammability of the obtained materials.

## 2. Materials and Methods

### 2.1. Raw Materials

Fresh and unrefined white mustard (*Sinapis alba*) seed oil (MO) was supplied by Perlo (Rzeszów, Poland) and used for synthesis of the new bio-polyols (MPs). The iodine value (IV) of this oil was 0.513 mol I_2_/100 g of oil, acid value (AV) was 4.320 mg KOH/g and the content of unsaturated fatty acids was 95.89% of all fatty acids. The following reagents were used for the first step of the synthesis, i.e., epoxidation of double bonds: 99.5% (glacial) acetic acid (AA), 30% hydrogen peroxide (HP) and 96% sulfuric acid (SA). All these reagents were supplied by Chempur (Piekary Śląskie, Poland). The next steps were different for each bio-polyol and the following reagents were used: 99% 2,2′-thiodiethanol (TDE, Merck, Darmstadt, Germany), boric acid (BA), xylene and 96% sulfuric acid (all from Chempur, Piekary Śląskie, Poland). Anhydrous magnesium sulfate (Chempur, Piekary Śląskie, Poland) was used for drying the bio-based polyol.

Synthesized bio-polyols containing sulfur and boron atoms and petrochemical polyether polyol Rokopol RF-551 (PCC Rokita S.A., Brzeg Dolny, Poland) were used in the mixture for production of rigid polyurethane/polyisocyanurate foams. The polyether polyol used in this research had a hydroxyl value of 420 mg KOH/g, an acid value of 0.05 mg KOH/g, a functionality of 4.5 and a molecular weight of 600 g/mol. Polymeric MDI—Purocyn B (Purinova Ltd., Bydgoszcz, Poland) was used as the isocyanate raw material. The catalysts for production of rigid polyurethane/polyisocyanurate foams was anhydrous potassium acetate (Chempur, Poland) used in 33% solution in diethylene glycol (DEG, Chempur, Piekary Śląskie, Poland) and 1,4-diazabicyclo[2,2,2]octane (DABCO, Alfa Aesar, Tewksbury, MA, USA) used also in 33% solution in DEG. Tegostab 8460 (Evonik, Essen, Germany) was a surfactant. Blowing agent was carbon dioxide obtained in reaction of distilled water and an excess of isocyanate raw material.

### 2.2. Synthesis of New Bio-Polyols

Three types of bio-polyols based on MO were obtained as part of the presented research. All synthesized polyols contained boron and sulfur atoms in their structure. Two syntheses were based on the developed method of obtaining sulfur-containing bio-polyols, which the authors described in [26,35,36]. In turn, the third method was a completely new approach to the synthesis of bio-polyols containing both of these heteroatoms.

#### 2.2.1. Synthesis of MP1 Bio-Polyol

**Scheme 1:**
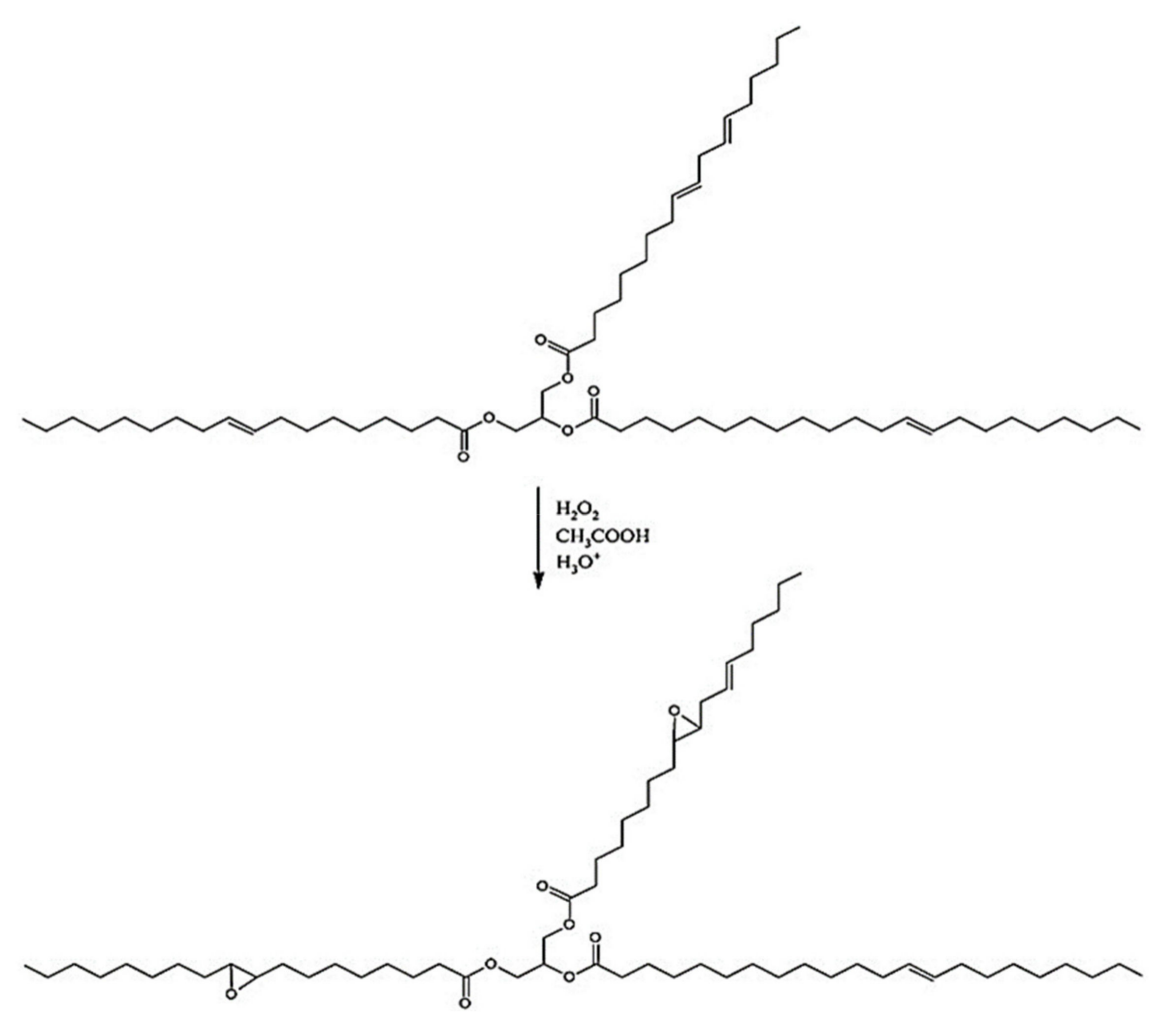
Epoxidation reaction of mustard seed oil.

The synthesis of MP1 bio-polyol was carried out in three steps. The first step was the epoxidation reaction of unsaturated bonds contained in white mustard oil. This step of bio-polyol synthesis was carried out in a glass reactor with a heating jacket equipped with a reflux condenser, dropping funnel, temperature sensor and mechanical stirrer. An amount of 389.86 g of MO, 120.70 g of glacial AA and 4.08 g of SA acid were loaded into reactor and heated to 40 °C.; 226.66 g of HP was gradually added at this temperature. After the addition of HP, the whole mixture was heated to 60 °C. The reaction was carried out for about 3 h. The molar ratio of the reactants calculated based on iodine value of MO was 1:1:1:0.02 for MO:AA:HP:SA. After reaction, two phases were observed—oil phase and water phase. The oil phase was separated from the water phase, and then, oil phase was washed with distilled water and dried with solid anhydrous magnesium sulfate. Epoxidized mustard seed oil (EMO) was obtained as a result of the first step of synthesis. The efficiency of the epoxidation process was lower due to the need to oxidize the remains of seeds and other substances contained in unrefined oil. However, such treatment removes these impurities from the epoxidized oil. Then, the oil was subjected to analytical tests to determine an epoxy value, which was necessary for the next step of the synthesis. The reaction scheme is shown in Scheme 1.

**Scheme 2:**
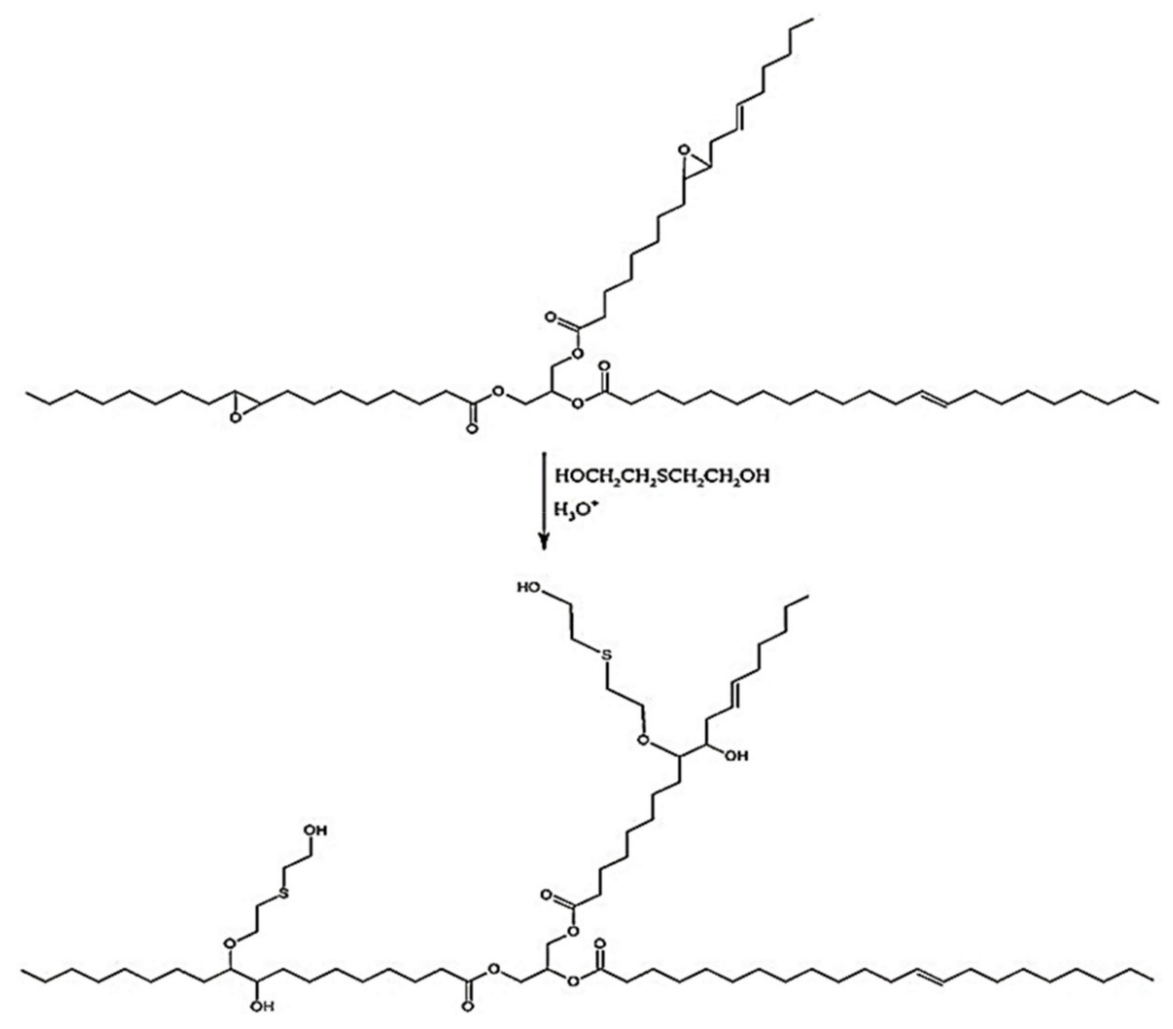
Opening of epoxy groups by 2,2′-thiodiethanol.

In the second step, 200.00 g of EMO (epoxy value—0.283 mol/100 g, reaction efficiency—55.17%), 69.86 g of 2,2′-thiodiethanol (TDE) and 0.58 g of the reaction catalyst—sulfuric acid—were loaded into a glass reactor. The reactor contained a heating jacket, a temperature sensor, a mechanical stirrer and a reflux condenser. The molar ratio of reactants calculated based on epoxy value of EMO was 1:1:0.01 for EMO:TDE:SA. Afterward, the whole mixture was heated to 120 °C. Reaction was carried out for five hours until the opening of all the epoxide groups by TDE. Mustard oil-based bio-polyols containing sulfur atoms were obtained after this step. Then, the catalyst was neutralized with an appropriate amount of sodium hydroxide solution. The remaining amount of water was distilled off under vacuum. The reaction scheme is shown in Scheme 2. The synthesized intermediate bio-polyol (containing sulfur atoms) was tested for its hydroxyl and acid values. The ratios of the reagents to the last step of the synthesis of MP1 bio-polyol were determined on the basis of its hydroxyl value.

**Scheme 3:**
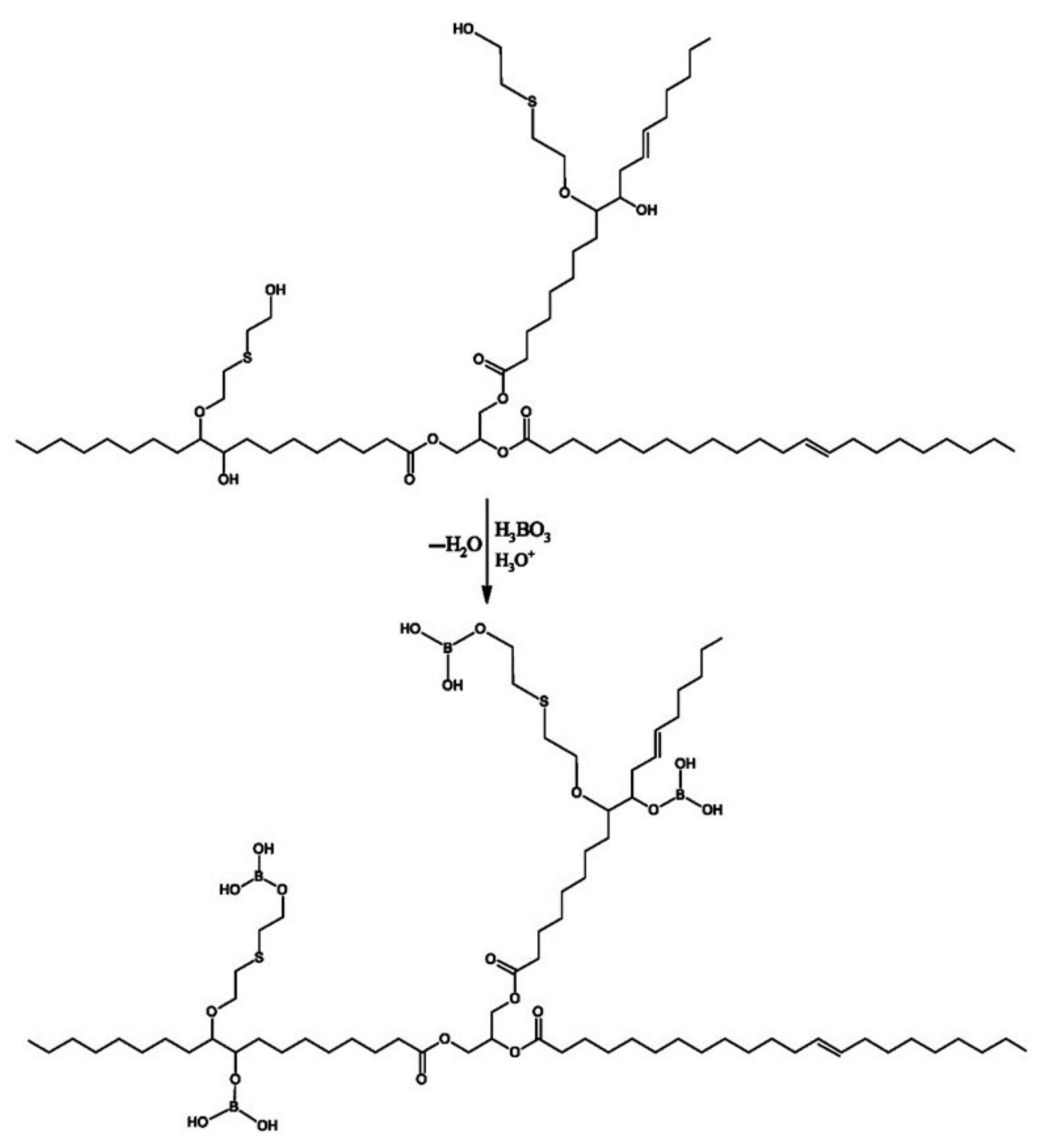
Synthesis of MP1 bio-polyol.

In the third step, boron atoms were incorporated into the structure of intermediate bio-polyol containing sulfur atoms. For this purpose, 100.00 g of the product of second step of synthesis (hydroxyl value—197.34 mg KOH/g) was reacted with 21.76 g of boric acid. The reaction was carried out in the form of azeotropic distillation in a reaction flask equipped with a Dean–Stark receiver with reflux condenser. The reaction of partial esterification of boric acid with intermediate bio-polyol (IB) was carried out in the presence of 2.50 g of an acid catalyst (sulfuric acid) and 100 mL of a solvent (xylene) forming an azeotropic mixture with the water formed in the reaction. The molar ratio of the reactants in the reaction was 1:1:0.07 for IB:BA:SA. The equimolar ratio of IB to BA meant that one hydroxyl group from bio-polyol would react with one OH group of boric acid. The reaction was carried out in the temperature range of 110–115 °C in which the water formed in the esterification reaction was distilled off. The end of the reaction was deemed to be the distillation of the stoichiometric amount of water that should be formed in this reaction. The esterification was carried out for about 3 h. After the reaction, the catalyst was neutralized with an appropriate amount of sodium hydroxide solution. A mixture of MP1 bio-polyol, xylene and residual water from catalyst neutralization was distilled under reduced pressure to remove the solvent and water from the final product. The obtained bio-polyol was subjected to further tests. The scheme of the MP1 bio-polyol synthesis reaction is shown in Scheme 3.

#### 2.2.2. Synthesis of MP2 Bio-Polyol

**Scheme 4:**
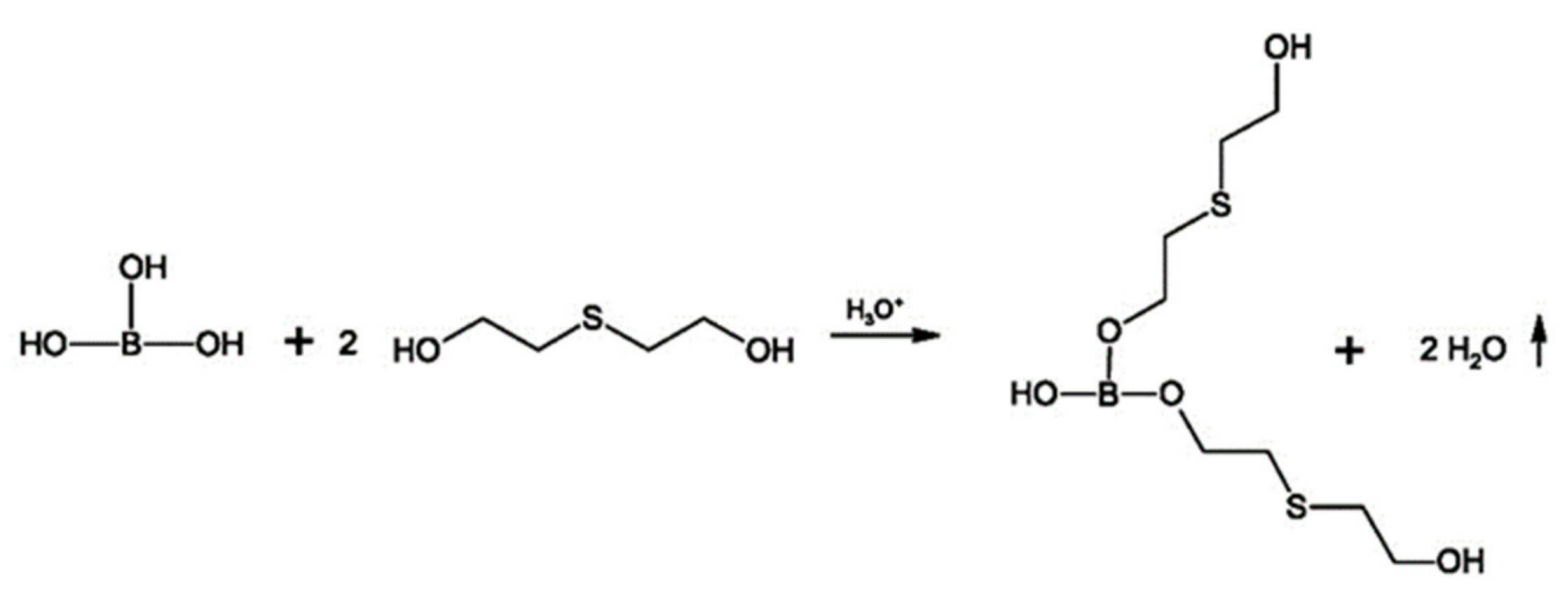
Synthesis di(2-(2-hydroxyethylsulfanyl)ethyl) borate (BD).

The synthesis of the MP2 bio-polyol was carried out in four steps. The first two steps were analogous to the synthesis of the MP1 bio-polyol and led to the obtaining of the intermediate bio-polyol containing sulfur atoms (IB). The third step in this synthesis consisted of the synthesis of di(2-(2-hydroxyethylsulfanyl)ethyl) borate [abbrev. BD]. For this purpose, 61.83 g of BA, 244.38 g of TDE, 7.15 g of SA and 200 mL of xylene were introduced into a reaction flask equipped with a mechanical stirrer, temperature sensor and a Dean–Stark receiver with reflux condenser. The molar ratio of the reactants was 1:2:0.07 for BA:TDE:SA. The reaction was carried out by azeotropic distillation, removing the water formed in the reaction of TDE with BA. The azeotropic mixture containing the water formed in the reaction was distilled at 113 °C until its stoichiometric amount was obtained. After the reaction, the catalyst was neutralized with an appropriate amount of sodium hydroxide solution. The obtained BD was distilled under reduced pressure until the xylene and residual water were completely removed. The scheme of the BD synthesis reaction is shown in Scheme 4.

**Scheme 5:**
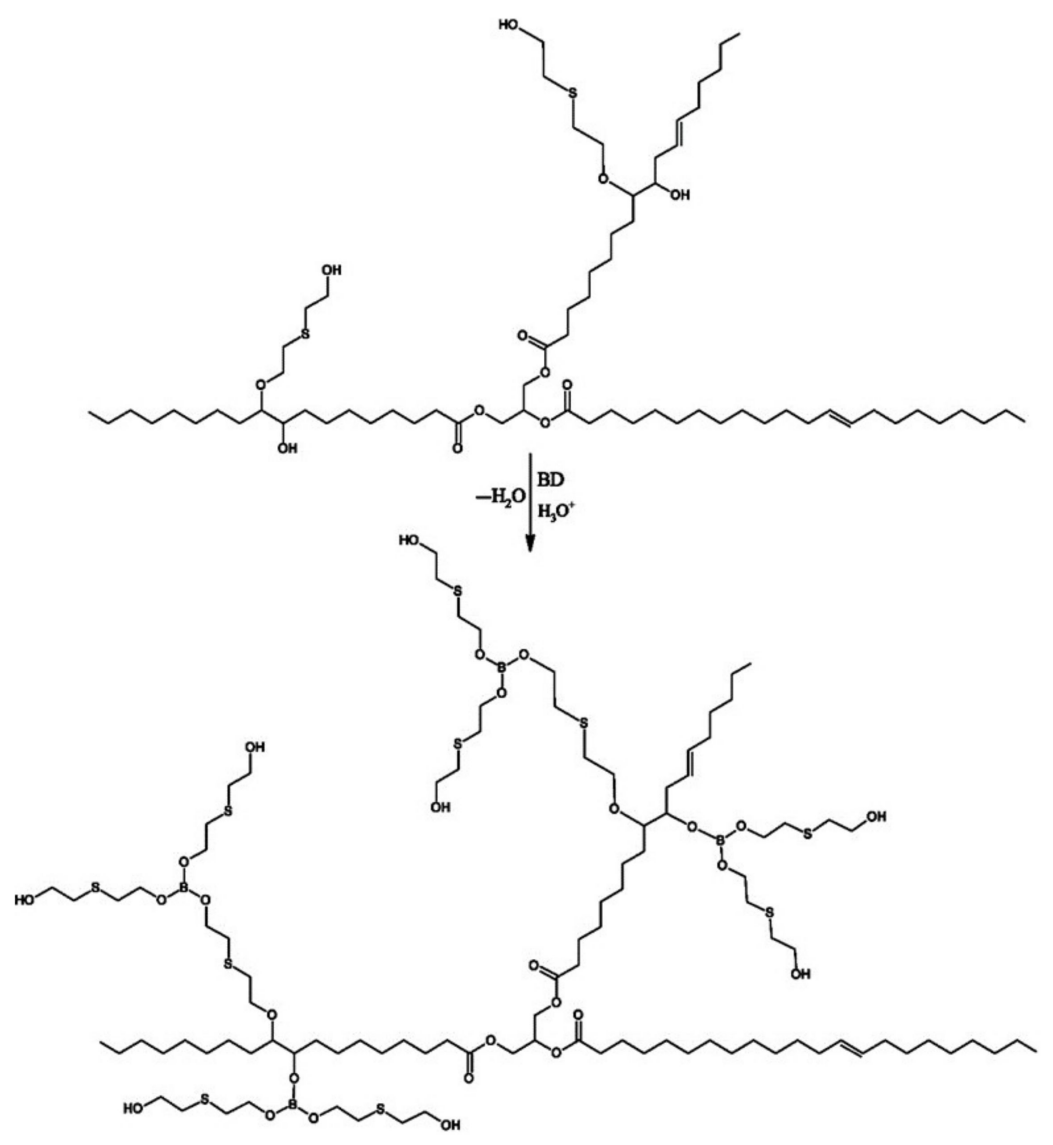
Synthesis of MP2 bio-polyol.

The obtained BD was used for the reaction with IB in the last step of the synthesis of MP2 bio-polyol. The reaction was again carried out by azeotropic distillation in a reaction system equipped with a Dean–Stark receiver with reflux condenser. Equimolar amounts of IB (100.00 g) and BD (94.57 g), and SA in the amount of 2.50 g, were introduced into the reaction flask. Additionally, 100 mL of xylene was added as a solvent-forming azeotrope with the water formed in reaction. The distillation was carried out at the temperature of 115 °C until the stoichiometric amount of water resulting from the amount of introduced reagents was obtained. After the reaction, the catalyst was neutralized with an appropriate amount of sodium hydroxide solution. Then, xylene and residual water were removed by distillation under reduced pressure. The end product was MP2 bio-polyol. The reaction scheme for the synthesis of MP2 polyol is shown in Scheme 5.

#### 2.2.3. Synthesis of MP3 Bio-Polyol

**Scheme 6:**
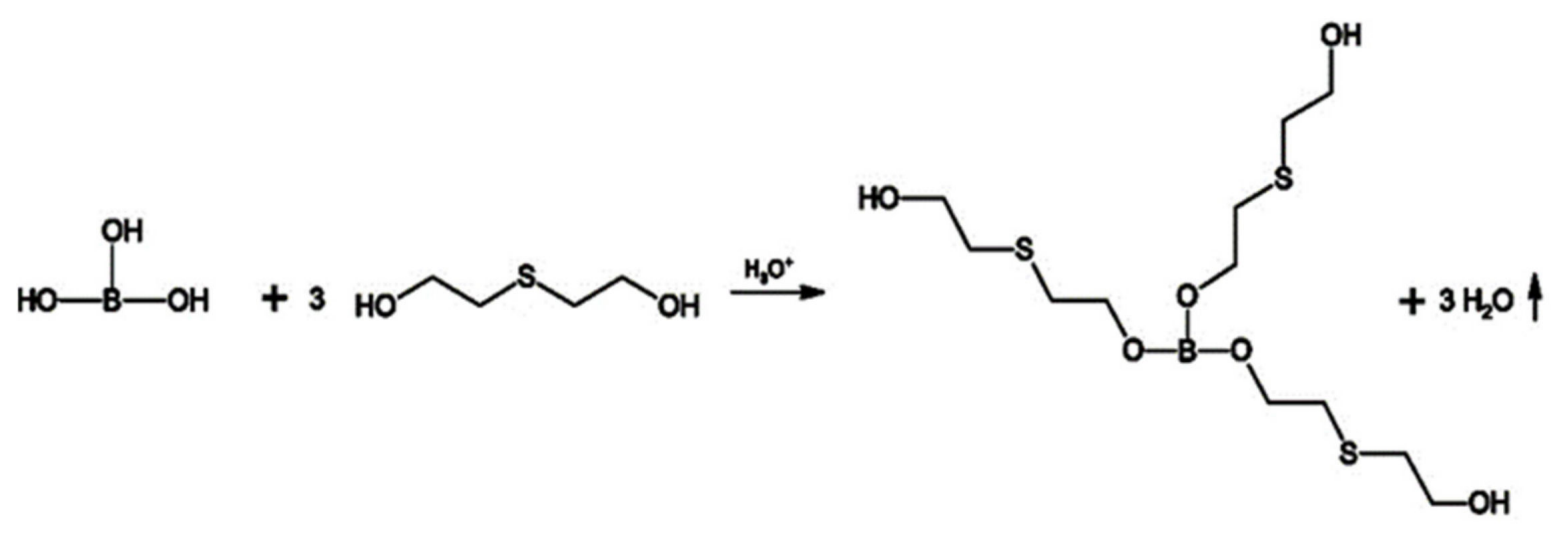
Synthesis of tri(2-(2-hydroxyethylsulfanyl)ethyl) borate (BT).

The synthesis of MP3 bio-polyol was carried out in three steps. The first was analogous to the synthesis of MP1 and MP2 bio-polyols. It led to obtaining epoxidized mustard oil (EMO). Independently of the first step, tri(2-(2-hydroxyethylsulfanyl)ethyl) borate [abbrev. BT] was synthesized. The reaction was carried out in the same way as the synthesis of BD. It differed only in the molar ratios of the reactants, which were 1:3:0.07 (61.83 g:366.57 g:7.15 g) for BA:TDE:SA. The scheme of the BT synthesis is shown in Scheme 6.

**Scheme 7:**
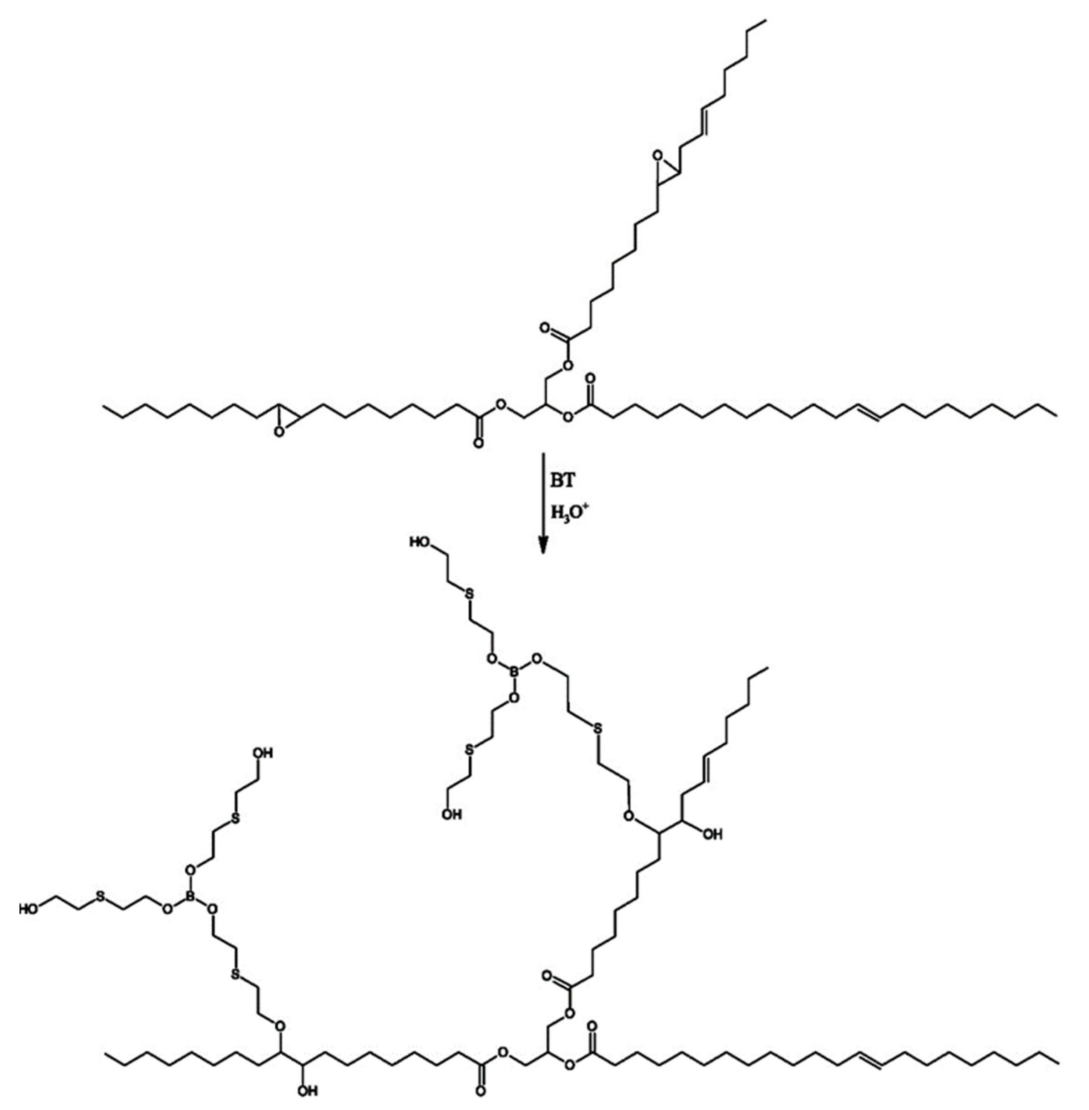
Synthesis of MP3 bio-polyol.

The obtained BT was used as an epoxy ring-opening agent of EMO. For the synthesis of MP3 bio-polyol, 100.00 g of EMO (epoxy value—0.283 mol/100 g), 105.96 g of BT and 0.29 g of SA were introduced into a reaction flask with a heating jacket equipped with a reflux condenser, a mechanical stirrer and a temperature sensor. The molar ratio of the reactants was 1:1:0.01 for EMO:BT:SA. The whole mixture was heated to 120 °C and the epoxy ring-opening reaction was started. The reaction was carried out for approximately four hours. The reaction was complete when the epoxy value of the bio-polyol was 0. The obtained MP3 bio-polyol was cooled to ambient temperature and neutralized with an appropriate amount of sodium hydroxide solution. The remaining amount of water was distilled off under vacuum. The scheme of the synthesis of MP3 bio-polyol is shown in Scheme 7.

### 2.3. Assessment of Bio-Polyol Properties

Physicochemical, analytical, spectroscopic and thermal tests were carried out on the new bio-polyols based on MO. These studies were aimed at determining the basic characteristics of the obtained compounds and comparing their properties.

#### 2.3.1. Physicochemical Tests of Bio-Polyols

State of matter, color and smell of obtained white mustard oil based-polyols were tested organoleptically. Viscosity of the bio-polyols was determined using a FungiLab (New York, NY, USA) digital rheometer at 20 °C (293 K). Density of bio-polyols was measured at 25 °C (298 K) in an adiabatic pycnometer according to PN-EN 92/C-04504. Value of pH was measured by Hanna Instruments (Woonsocket, RI, USA) laboratory pH-meter (ORP/ISO/°C).

#### 2.3.2. Analytical Tests of Bio-Polyols

The hydroxyl value and the acid value were tested according to Purinova Ltd. Standards—WT/06/07/PURINOVA. The water content was checked by the Karl Fischer method according to PN-81/C-04959. Content of carbon, hydrogen, nitrogen, sulfur and oxygen in new bio-polyols was measured by Vario EL III CHNSO analyzer (Elementar Analysensysteme GmbH, Langenselbold, Germany). Content of boron in new compounds was analyzed by ICP-MS spectrometer (NexION 300D, Perkin-Elmer, Waltham, MA, USA). The number-average molecular weight (M_n_), the weight-average molecular weight (M_w_) and dispersity (D) of the bio-polyols were determined by Gel Permeation Chromatography (GPC) using a Knauer chromatograph (Berlin, Germany). The device was equipped with refractometer, detector and thermo-stated columns. Functionalities of bio-polyols were calculated on the basis of hydroxyl value and number-average molecular weight.

#### 2.3.3. Spectroscopy Tests

The bio-polyols containing boron and sulfur heteroatoms were tested in Fourier-transform infrared (FTIR) spectroscopy by Nicolet iS20 spectrophotometer (Thermo Fisher Scientific, Waltham, MA, USA). The measurement was made in a spectral range from 400 to 4000 cm^−1^. Bio-polyols were also tested in proton and carbon nuclear magnetic resonance spectroscopy (^1^H NMR and ^13^C NMR) using a Bruker NMR Ascend III spectrometer (Billerica, MA, USA) with a frequency of 400 MHz. Deuterated chloroform was used as a solvent. Time of NMR acquisition was 4.000 s.

#### 2.3.4. Differential Scanning Calorimetry

Bio-polyols based on mustard oil were tested by differential scanning calorimetry (DSC). The measurement was made using a Netzsch DSC 204 F1 device in a nitrogen atmosphere. Temperature of measurement was from −40 to 180 °C. The weight of the sample used for the test was several milligrams. The test was performed in one measurement cycle.

### 2.4. Preparation of Rigid Polyurethane/Polyisocyanurate Foams Based on MPs Bio-Polyols

Development of the rigid polyurethane/polyisocyanurate foam formulations modified by bio-polyols containing boron and sulfur atoms required experimental tests to determine the most advantageous composition of all additives (surfactant, catalyst, blowing agent, etc.). The basis for determining the amounts of polyol raw materials was always the hydroxyl values of petrochemical polyol Rokopol RF-551 and MPs bio-polyols. On this basis, equivalent amounts of polyols were calculated according to Equation (1):

(1) $$Eq_{OH}=\frac{Eq_{KOH}}{HV}$$

where: *Eq_OH_*—mass of mustard oil-based bio-polyol (g); *Eq_KOH_*—chemical equivalent of hydroxyl groups in potassium hydroxide, which equals its molar mass—56,100 (mg/mol); *HV*—hydroxyl value of petrochemical polyol or synthesized bio-polyol (mg KOH/g).

The amount of isocyanate used in preparation of rigid polyurethane/polyisocyanurate foams was calculated based on the chemical equivalent, which is correlated with percentage value of free *NCO* groups (*Eq_NCO_*). This parameter referred to a mass of isocyanate raw material corresponding to chemical equivalent of free isocyanate groups. This parameter was calculated based on Equation (2):

(2) $$Eq_{NCO}=\frac{4200}{{\%}_{NCO}}$$

Ratio of *NCO* to OH equivalents (*Eq_NCO_*:*Eq_OH_*) in this case was 3.7:1. The excess isocyanate feedstock compared to the polyol feedstock is necessary because three major reactions will take place in the reaction system: reaction between the *NCO* and OH groups to form a urethane bond, the trimerization of the three *NCO* groups to the isocyanurate ring and reaction between an excess of isocyanate raw material and distilled water to generate a blowing agent—carbon dioxide. In the developed formulations, the sum of *Eq_OH_* of petrochemical polyol and MPs bio-polyols was 1.0. The most favorable content of additives was the amine catalyst, 1.0 wt.%; the trimerization catalyst, 2.5 wt.%; chemical blowing agent, 0.7 *Eq_OH_* (constant value in all foams); and a foam stabilizer, 1.7 wt.%. All percentages were in relation to the sum of the masses of polyols and isocyanate raw materials.

Newly synthesized bio-polyols containing boron and sulfur atoms were used in the production of rigid polyurethane/polyisocyanurate foams. The bio-polyols were added into the formulation gradually (every 0.1 chemical equivalent of OH groups) by partially replacing petrochemical polyol (Rokopol RF-551). Modifications of the formulation were carried out until the addition of an amount of bio-polyols did not significantly deteriorate the process of obtaining a rigid foam. In first case, this amount was 0.1 *Eq*. In second and third cases, this amount was 0.5 *Eq*. The rigid polyurethane/polyisocyanurate foam formulations are presented in Table 1.

Rigid polyurethane/polyisocyanurate foams were obtained in a laboratory scale using the one-step method. A system consisting of two components was used during the preparation of the foams. First component was a mixture of appropriate amounts of petrochemical polyol, bio-polyol, two catalysts, surfactant and distilled water. Second component was isocyanate raw material—Purocyn B. Both components were mixed by mechanical stirrer for 10 s in an appropriate mass ratio. After mixing, reactant mixture was poured into a cuboidal metal mold. The used mold had internal dimensions of 25 cm × 25 cm × 30 cm. Due to the fact that the mold was open on one side, the foam could rise freely.

Twelve types of foams were obtained in this research: a reference foam without bio-polyols, one foam with MP1 bio-polyol, five foams with MP2 bio-polyol (MP2 series) and five foams with MP3 bio-polyol (MP3 series). The MP series foams contained an increasing content of appropriate bio-polyol by 0.1 chemical equivalent relative to each other. The production of each rigid polyurethane/polyisocyanurate foam was repeated twice. After removal from the mold, all obtained foams were thermostated in a forced circulation dryer for 4 h at 120 °C. Then the foams were cut into standardized samples and subjected to appropriate tests. Based on the measurements of the geometric volume and weight of the samples, their apparent density was determined in accordance with the ISO 845:2010 standard. Results of apparent density of foams modified by new bio-polyols are presented in Table S1 in Supplementary Materials.

### 2.5. Flammability Tests of Rigid Polyurethane/Polyisocyanurate Foams Modified by Bio-Polyols Containing Sulfur and Boron Atoms

Four flammability tests were performed on the new rigid polyurethane/polyisocyanurate foams: vertical combustion test (determination of the residue after combustion); horizontal combustion test (determination of the behavior of the sample after subtraction of the flame), limiting oxygen index (LOI) test and cone calorimeter test. Vertical combustion test was made according to ASTM D3014-73 and horizontal combustion test according to ISO 3582:2002/A1:2008.

Rigid polyurethane/polyisocyanurate foams modified by bio-polyols containing boron and sulfur heteroatoms were tested by cone calorimeter (Fire Testing Technology, East Grinstead, UK) according to ISO 5660:2015 standard. All samples were subjected to a controlled heat flux (50 kW/m^2^) to analyze fire behavior and smoke release. The following parameters were measured as a result of this test:

- TTI—time to ignition, (s);
- THR—total heat release from the surface unit of the analyzed material, (MJ/m^2^);
- MLR—mass loss rate of sample/burning rate, (g/m^2^·s);
- HRR—heat release rate from the sample during combustion, (kW/m_2_);
- t_HRRmax_—time to reach the maximum value of HRR (HRR_max_), (s);
- CO, CO_2_—emission of CO and CO_2_, respectively (kg/kg);
- TSR—total smoke release, (m^2^/m^2^).

The limiting oxygen index of obtained foams was determined according to ASTM D 2863-1970 standard. For this measurement, Concept Equipment (Poling, West Sussex, UK) apparatus was used. It allowed us to determine the value of this parameter with an accuracy of 0.1%. The percentage limits of O_2_ in a mixture consisting of O_2_ and N_2_ sufficient to sustain the combustion of the sample were determined.

## 3. Results and Discussion

### 3.1. Physicochemical Tests of Bio-Polyols

Three new bio-polyols (MP1-MP3) containing boron and sulfur heteroatoms were obtained as a result of the performed syntheses. First, the obtained compounds were subjected to basic organoleptic evaluation in order to determine their physical state, color and smell. Then, the bio-polyols were subjected to a series of physicochemical tests to accurately characterize their properties, such as density, viscosity and pH. The obtained results of the research on the physicochemical properties of the synthesized bio-polyols are presented in Table 2.

The color of the synthesized bio-polyols was strongly dependent on the color of the used white mustard seed oil. The natural color of this oil can range from yellow to light-brown [37]. In the case of the products synthesized in this research, one bio-polyol (MP1) was a brown solid (at 20 °C), and two bio-polyols (MP2 and MP3) were liquids of light-orange and orange color, respectively. White mustard seed oil is characterized by a delicate mustard smell, which was completely lost in the MP1 polyol. On the other hand, the MP2 and MP3 polyols had a slightly perceptible sulfuric smell. This resulted from the use of 2,2’-thiodiethanol and its derivatives in the form of boric acid esters as epoxy ring opening agents in epoxidized oil and esterifying agent. The densities of the MP2 and MP3 bio-polyols tested at 25 °C were similar and were 1.09 and 1.10 g/cm^3^, respectively. The slight difference was due to the application of various methods of introducing boron and sulfur atoms into bio-polyols. This difference resulted in a different structure of the bio-polyol molecules. On the other hand, the density of the MP1 bio-polyol was the highest of all and was 1.19 g/cm^3^. This was due to the strong interaction of the OH groups combined with the boron atom [27].

A very important parameter pertaining to the use of polyols for the synthesis of polyurethane materials is viscosity. Significant differences in the viscosity of the obtained polyols based on mustard oil were noted during the tests. In the case of the MP1 bio-polyol, it was solid at 20 °C and it was not possible to measure its viscosity. This may result in difficult processing of this raw material or the need to heat it before use. On the other hand, the viscosities of the MP2 and MP3 bio-polyols were 9.400 and 8.800 mPa·s, respectively. The decrease in viscosity was also caused by the difference in the structures of the bio-polyols resulting from the use of different reactions to the incorporation of boron and sulfur heteroatoms. Heteroatoms were introduced into the MP2 bio-polyol in the reaction of the intermediate-polyol containing sulfur atoms with di(2-(2-hydroxyethylsulfanyl)ethyl) borate (BD). In turn, MP3 was obtained as a result of the epoxy ring-opening reaction of epoxidized mustard oil with tri(2-(2-hydroxyethylsulfanyl)ethyl) borate (BT). The use of BD and BT for the synthesis of MP2 and MP3 bio-polyols resulted in obtaining more branched structures. The use of BD resulted in obtaining a more branched structure than in the case of using BT. This was due to the fact that BD esterified all the hydroxyl groups of the intermediate-polyol. It was built into both the primary hydroxyl groups derived from 2,2’-thiodiethanol, which previously opened epoxy rings, and the secondary hydroxyl groups on the fatty acid chains. As a result of the esterification reaction, a bio-polyol was obtained that had only primary hydroxyl groups derived from the incorporated BD molecules. In turn, the use of BT as an epoxy ring-opening agent resulted in the incorporation of one BT molecule for each epoxy group. A consequence of this reaction was the formation of a secondary hydroxyl group on the fatty acid chain. Longer chains and their branched structure increase the viscosity of polyols [19,38]. The method of synthesis also had a significant impact on polyol parameters such as hydroxyl value and functionality, which also influenced the viscosity of the bio-polyols. Typically, the polyols with lower functionality have lower viscosities [38]. In industrial practice, viscosities above 5000 mPa·s are not recommended from an economic point of view [39].

Measurement of pH showed that the value of this parameter was in the range of 4.5–7.1 for all compounds. This meant that the obtained products were acidic (MP1) or neutral (MP2 and MP3). The high value of this parameter for the MP1 bio-polyol was due to the fact that incompletely substituted boric acid was incorporated into the polyol molecule. The presence of B-OH groups capable of hydrolysis significantly decreased the pH value.

### 3.2. Analytical Tests of Bio-Polyols

Very important parameters of polyols from the point of view of their use in the synthesis of polyurethane materials are hydroxyl value, acid value and water content. The obtained test results are presented in Table 3.

The hydroxyl value is of great importance when using polyol raw materials in formulation for the production of PU materials. It influences the properties of the obtained material (e.g., mechanical strength) and its type (e.g., rigid and flexible foam, elastomer, etc.) [40]. For polyol MP1, the hydroxyl value was equal to the acid value (AV) and was 55.25 mg KOH/g. This meant that the *HV* of the MP1 polyol was closely related to the OH groups derived from unsubstituted boric acid which were not acylated during the *HV* determination. On the other hand, MP2 and MP3 bio-polyols had *HV*s at a similar level. They were 188.34 and 178.42 mg KOH/g, respectively. This small-but-significant difference in the values of this parameter resulted from the use of two different methods of synthesizing these bio-polyols. The use of BD as an esterifying agent resulted in a higher *HV* of the MP2 bio-polyol. Namely, the opening of the epoxy ring by 2,2’-thiodiethanol resulted in the formation of two hydroxyl groups from each epoxy group of the intermediate-polyol. As a result of the esterification reaction, additional BD molecules were incorporated into each OH group. This meant that, finally, four primary hydroxyl groups were obtained from one epoxy group of epoxidized oil (first intermediate product). In the case of the MP3 bio-polyol, the use of BT as an epoxy ring-opening agent led to the formation of three OH groups (two primary and one secondary). This difference in the chemical structures of the two bio-polyols resulted in a difference in the hydroxyl value.

Another important parameter of polyol raw materials is the acid value. The obtained compounds were characterized by different AVs. The MP1 bio-polyol had the highest value (55.25 mg KOH/g). Such a high AV was the result of the presence of incompletely substituted boric acid, which resulted from the assumptions of the conducted synthesis. The presence of free OH groups on the boron atom was also confirmed by the pH value of this bio-polyol, which was 4.5. The low pH value also meant that these OH groups were able to hydrolyze and generate H+ ions responsible for the acidity of the bio-polyol. In the case of the MP2 and MP3 bio-polyols, the acid values were much lower and were 2.42 and 1.13 mg KOH/g, respectively. For the synthesis of these compounds, boric acid di- and tri-ester were used. No free B-OH groups were present in these bio-polyols after the reaction. The AVs in these polyols resulted mainly from the presence of free or hydrolyzed fatty acids occurring naturally in vegetable oils [41,42].

The water content of the bio-polyols obtained by three different methods ranged from 0.05 to 0.1 wt.%. In the case of the MP1 and MP2 bio-polyols, water formed during the esterification reaction of boric acid or BD with the hydroxyl group of the intermediate-polyol. The amount of water at this level was related to carrying out the reaction in the form of azeotropic distillation in which the water formed in the reaction was distilled from the system. Additionally, the bio-polyols were further purified from the solvent (xylene) by distillation under reduced pressure. In turn, the water content of the MP3 polyol could have been derived from BT. BT was also obtained by the azeotropic distillation method in which water was removed from the reaction system. It is worth noting that the water content in all new bio-polyols was within the range acceptable for the synthesis of polyurethane materials [43].

The obtained bio-polyols were tested for their elemental composition in order to determine the share of individual elements in their molecules (with particular emphasis on the content of sulfur and boron). The obtained results are presented in Table 4.

The analysis of the elemental composition of the synthesized bio-polyols showed that the sulfur and boron heteroatoms were successfully incorporated into the structure of each. The highest boron content was noted in the case of the MP1 bio-polyol; it was 4.05%. This resulted from the complete conversion of all hydroxyl groups with boric acid. The sulfur content in this bio-polyol was also relatively high (5.08%). It should be remembered that the molecular weight of sulfur is almost three times higher than the molecular weight of boron. In the other polyols, the boron content was already lower and was 1.82% for MP2 and 0.80% for MP3. In turn, the sulfur content was higher in the case of MP1 and was 7.44% for MP2 and 5.49% for MP3. This was mainly due to the use of boric acid esters for their synthesis. Each of the esters had substituted at least two molecules derived from 2,2’-thiodiethanol. This resulted in an increase in the sulfur content of the MP2 and MP3 bio-polyols and a simultaneous decrease in the amount of other elements (e.g., boron). The introduction of heteroatoms to the bio-polyol molecule (such as boron) is important in reducing the flammability of polyurethane materials based on these bio-polyols. The increase in the content of such elements often results in a decrease in flammability and a decrease in the emission of toxic smoke generated during the combustion of these materials [44,45].

The obtained bio-polyols containing boron and sulfur atoms were also analyzed by the GPC method. This was performed to determine the number-average molecular weight (*M_n_*), weight-average molecular weight (M_w_) and dispersity (D). The results of this test are presented in Table 5. Additionally, the functionalities (*f*) of the synthesized bio-polyols were calculated on the basis of *HV* and *M_n_* from Equation (3):

(3) $$f=\frac{M_{n}\cdot HV}{56,100}$$

The GPC analysis showed that the *M_n_* of bio-polyols obtained by three different methods differed from each other and were 2654 g/mol for the MP2 bio-polyol, 2321 g/mol for the MP3 bio-polyol and 1265 g/mol for the MP1 bio-polyol. This difference is a consequence of obtaining different chemical structures resulting from the use of different methods of preparation. The highest value of *M_n_* was noted for MP2. In MP2, one molecule of 2,2’-thiodiethanol was introduced on one epoxy ring. As a result of this reaction, two hydroxyl groups were obtained—one at the fatty acid chain and the other at the end of the chain from 2,2’-thiodiethanol. Then, each of these groups reacted with BD. In total, five molecules of 2,2’-thiodiethanol and two molecules of boric acid for each epoxy ring were used for the synthesis of MP2. The presence of additional chains made this molecular weight the highest. On the other hand, in the MP3 bio-polyol was a secondary hydroxyl group instead of additional chains. This meant that there were three molecules of 2,2’-thiodiethanol and one molecule of boric acid per epoxy ring. Consequently, the *M_n_* of MP3 was lower than that of MP2. The lowest value of this parameter was noted for the MP1 bio-polyol. During the synthesis of this bio-polyol, for each epoxy ring, there was one molecule of 2,2′-thiodiethanol and two molecules of boric acid. This was definitely less than in the case of the MP2 and MP3 polyols. The GPC analysis showed that the M_w_ value was the highest for the MP1 bio-polyol; it was 4131 g/mol. Such a high value of weight-average molecular weight could have resulted from the dimerization of the molecules. The high value could have occurred as a result of the esterification reaction between boric acid incorporated into one molecule and the unsubstituted hydroxyl group of the other molecule. A confirmation of the high molecular weight distribution in the MP1 polyol was the high dispersity (D) value, which in this case was 3.27. The M_w_ values of the MP2 and MP3 bio-polyols were 3998 and 2887 g/mol, respectively. These values confirmed that the use of BD and BT for synthesis was the correct way to incorporate boron and sulfur atoms into the bio-polyol structures. Both methods of synthesis led to obtaining compounds with low molecular weight distributions. This was confirmed by the low dispersity values, which were 1.51 for MP2 and 1.24 for MP3. Moreover, the dispersity of the bio-polyols presented in this article was also due to the fact that they were obtained from white mustard seed oil. Vegetable oils are not one particular chemical compound with a constant molecular weight. They are a mixture of various fatty acid esters with different molecular weights.

Functionality is a very important parameter characterizing polyols in terms of their use for the synthesis of various types of polyurethane materials. The functionality of MP1 was low (1.24). This confirmed the formation of oligomers in the bio-polyol molecules, where the OH groups of incorporated boric acid were esterified by hydroxyl groups of another bio-polyol molecule. As a consequence, the amount of free OH groups and the functionality of the molecule were reduced. The MP2 and MP3 bio-polyols were characterized by relatively high functionality; it was 8.90 and 7.38, respectively. The difference between them was due to the fact that different methods of synthesis were used. In the case of the synthesis of the MP2 bio-polyol, BD reacted with the OH group at the end of the 2,2′-thiodiethanol chain and the OH group in the fatty acid chain. The incorporation of each into the structure of the bio-polyol resulted in the formation of two new primary OH groups. In the case of the MP3 bio-polyol, a lower functionality was noted than in the MP2 bio-polyol. This was due to the different roles of BT in comparison with BD. BT was a ring-opening agent, not an esterification agent for hydroxyl groups. Therefore, both boric acid ester structures and unsubstituted hydroxyl groups on the fatty acid chains were present in the structure of the MP3 bio-polyol. It is worth noting that the higher the functionality, the more prone the polyol is to cross-linking during the synthesis of polyurethane materials (e.g., rigid foams), improving, for example, their mechanical properties. It is worth noting that higher polyol functionality promotes higher cross-linking during the synthesis of polyurethane materials (e.g., rigid foams). Higher cross-linking improves mechanical properties [46].

### 3.3. Spectroscopy Tests of Bio-Polyols

**Figure 1:**
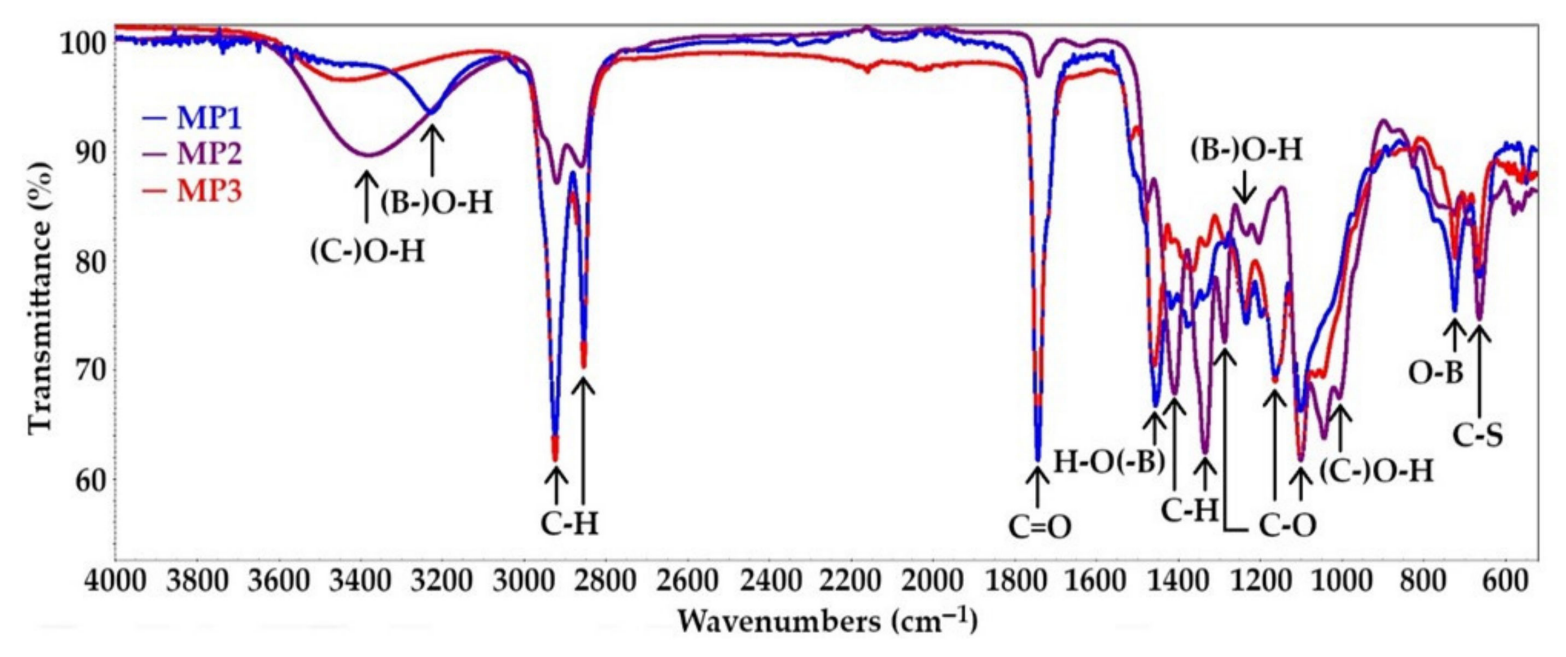
FTIR spectra of obtained bio-polyols.

**Figure 2:**
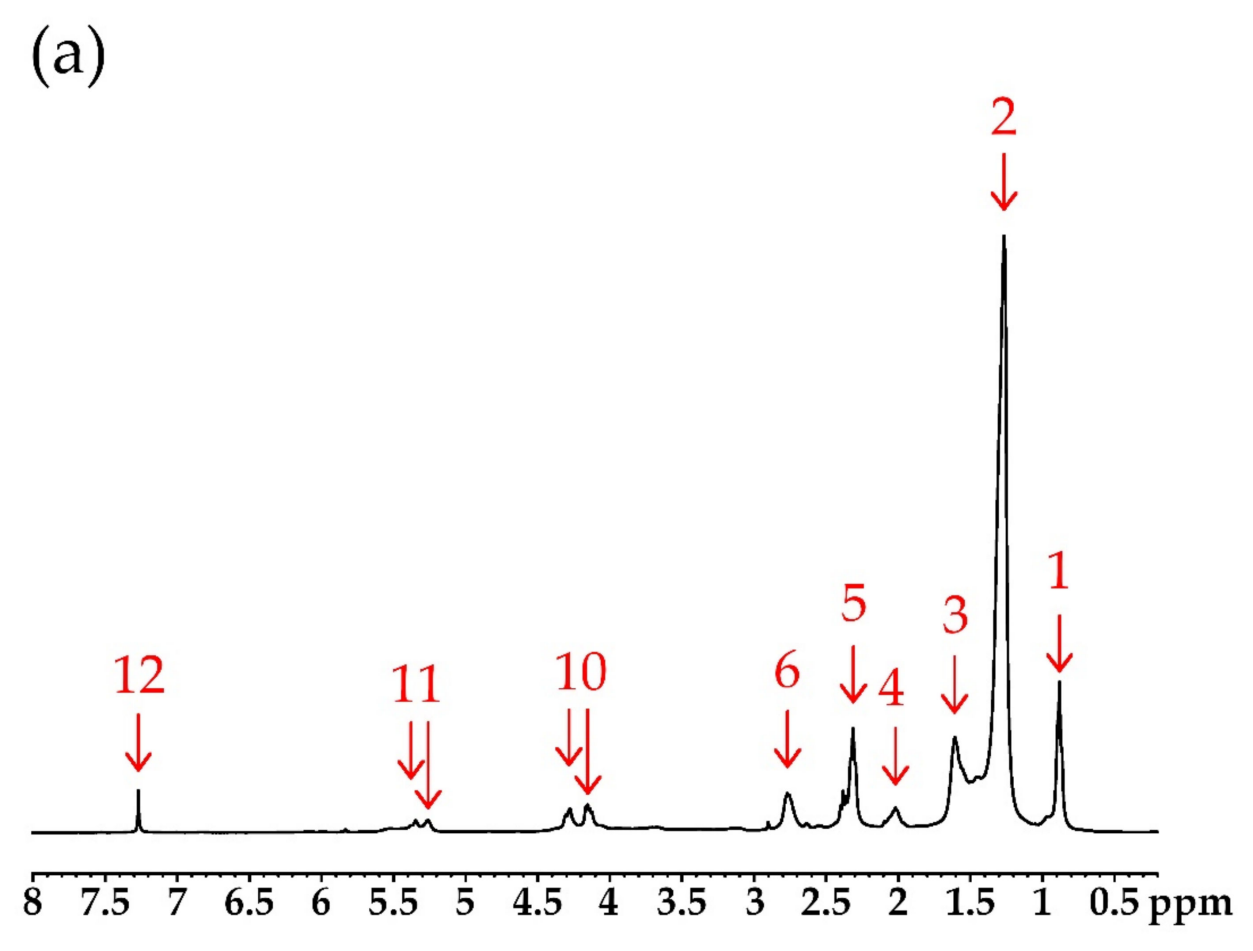

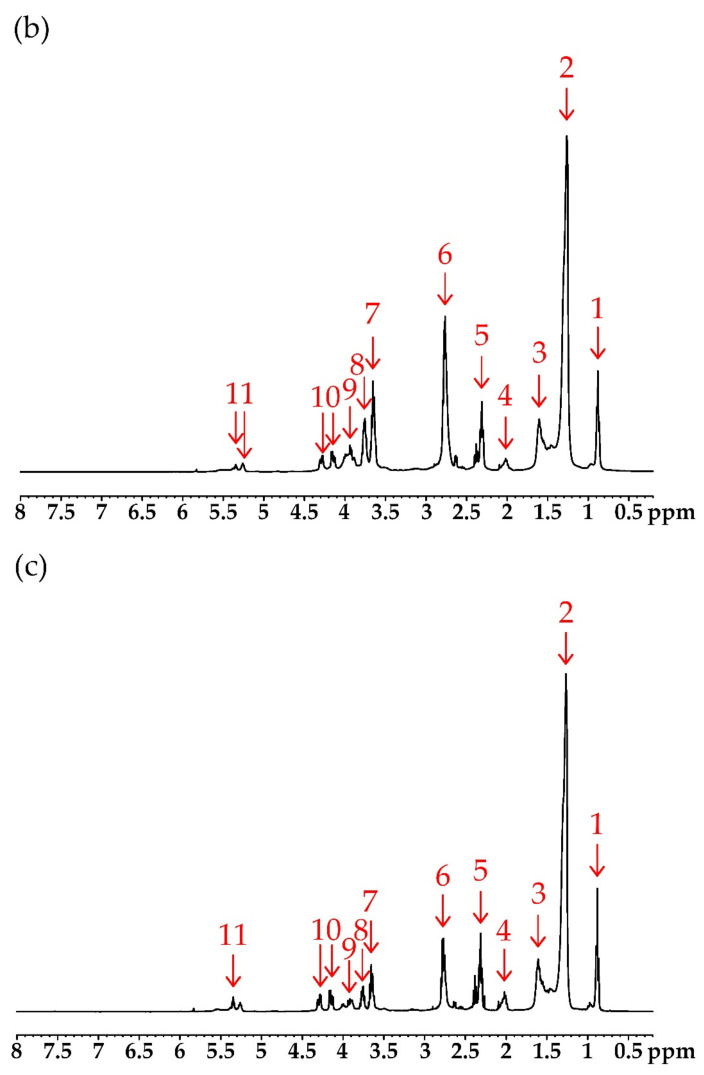
^1^H NMR spectra of obtained bio-polyols: (**a**) MP1, (**b**) MP2 and (**c**) MP3 (description of numbers in the Table 6).

**Figure 3:**
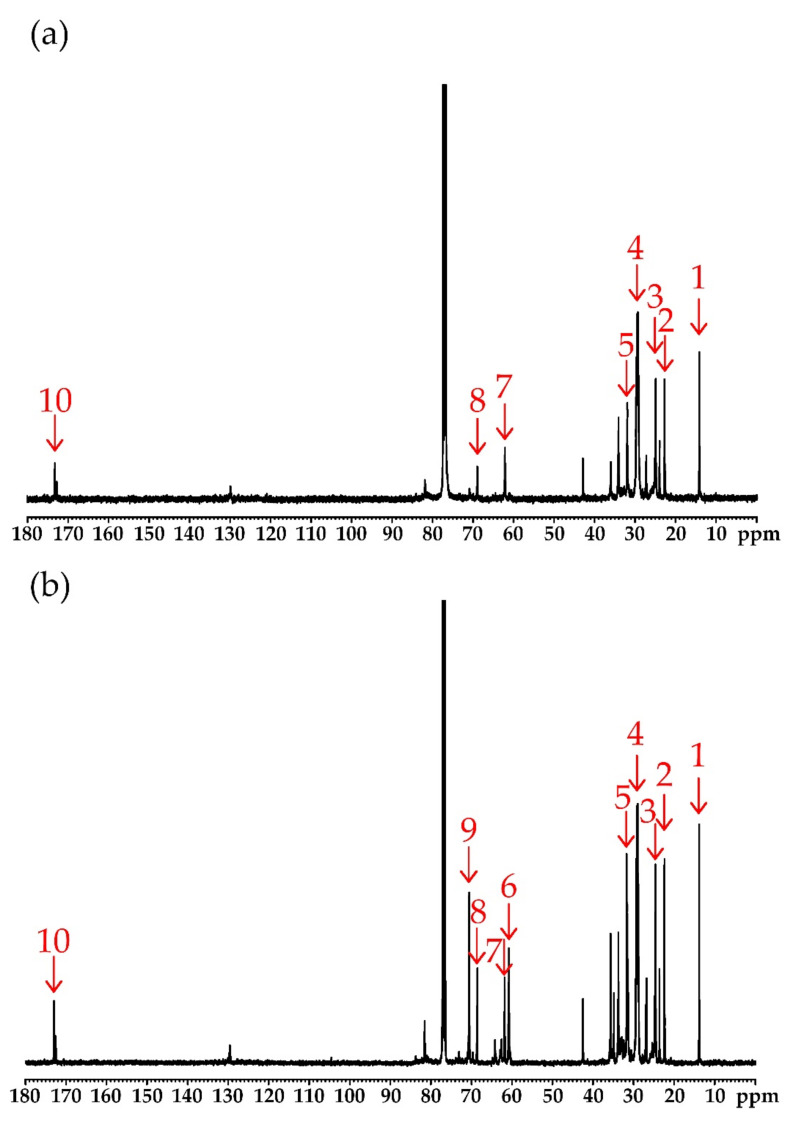

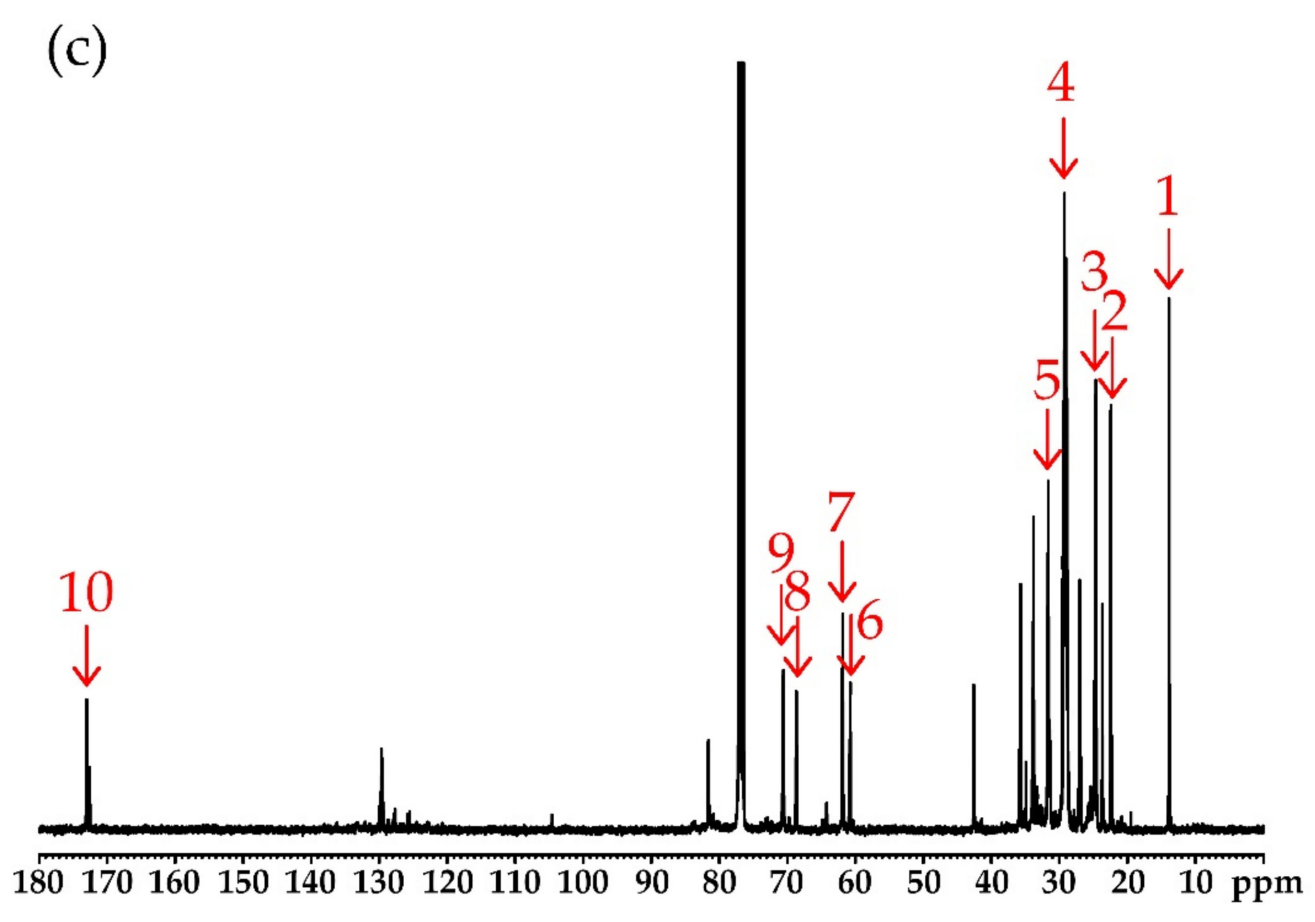
^13^C NMR spectra of obtained bio-polyols: (**a**) MP1, (**b**) MP2 and (**c**) MP3 (description of numbers in the Table 7).

When new chemical compounds are obtained, it is necessary to determine their chemical structure. Therefore, all three new bio-polyols were tested by FTIR, ^1^H and ^13^C NMR spectroscopy. The obtained spectra are shown in Figure 1, Figure 2 and Figure 3. All obtained spectra were interpreted on the basis of the spectroscopic spectra database—SDBS [47].

Analysis of the FTIR spectra of bio-polyols containing boron and sulfur heteroatoms showed that all newly synthesized compounds have characteristic groups for products obtained from vegetable oils. The bands of stretching vibrations at 2925 and 2870 cm^−1^ and the bands of deformation vibrations at 1460 and 1380 cm^−1^ belonged to C-H bonds from the -CH_2_- and -CH_3_ groups, respectively. The intense band of stretching vibrations at 1740 cm^−1^ belonged to the carbonyl group (C=O), while the bands at 1250, 1160 and 1099 cm^−1^ belonged to the C-O bonds of the ester group. In addition, all spectra also showed the presence of a band of stretching vibration at 655 cm^−1^ belonging to the C-S bond. This band was due to the fact that the chains derived from 2,2’-thiodiethanol were incorporated into the structure of the bio-polyols. A characteristic band at 781 cm^−1^ was also noted in all spectra. It belonged to the B-O bond, which indicated that the boron atoms had been incorporated into the bio-polyol molecule. The main difference in the discussed spectra was the bands of stretching vibrations belonging to the O-H bond. In the case of the MP1 bio-polyol, the occurrence of these bands was at different wavenumbers than in the case of the MP2 and MP3 bio-polyols. The bands of stretching vibrations belonging to this bond occurred at 3210 cm^−1^, and the bands of deformation vibrations occurred at 1475 and 1328 cm^−1^. For MP2 and MP3, the bands of stretching vibrations of the O-H bonds occurred at 3405 cm^−1^, and deformation vibrations occurred at 1034 cm^−1^. This difference was mainly due to the fact that the OH groups in the MP1 bio-polyol were directly combined with the boron atoms, while the OH groups in MP2 and MP3 were combined with the carbon atoms [48].

Analysis of the ^1^H NMR spectra (Figure 2) of bio-polyols showed the presence of characteristic chemical shifts. All interpreted chemical shifts are presented in Table 6 [20,35,49,50].

Analysis of the ^13^C NMR spectra (Figure 3) of bio-polyols showed the presence of characteristic chemical shifts. All interpreted chemical shifts are presented in Table 7 [20,35,49,50].

All spectroscopic analyses confirmed the assumed chemical structure of the newly synthesized bio-polyols contained sulfur and boron heteroatoms.

### 3.4. Differential Scanning Calorimetry of Bio-Polyols

**Figure 4:**
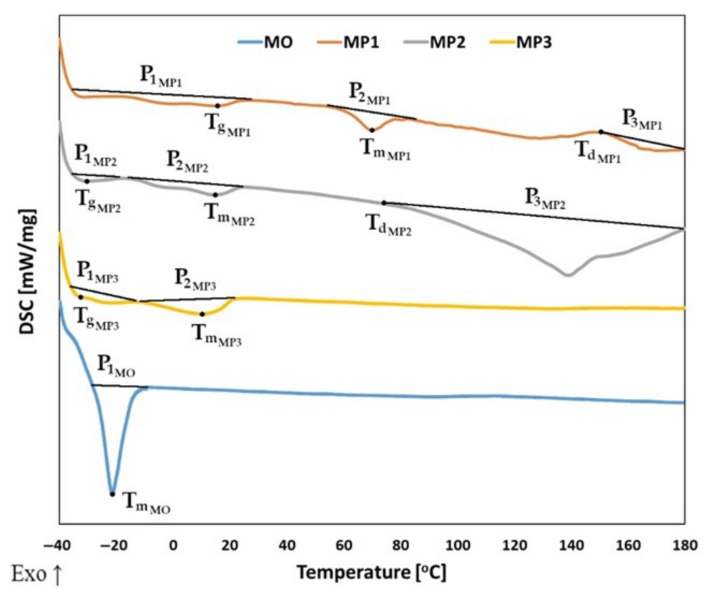
DSC curves of bio-polyols.

White mustard oil and three bio-polyols containing boron and sulfur atoms were subjected to DSC analysis to determine the possible changes occurring during heating. In the production process of polyurethane materials, a very large amount of heat is often released. Examining the possibility of endothermic or exothermic changes is important when new bio-polyols are used to obtain polyurethane/polyisocyanurate foams. The absence of thermal changes could mean the stability of these compounds in the production of PU materials [6]. The DSC thermograms of white mustard oil and bio-polyols containing heteroatoms are shown in Figure 4.

On the DSC thermogram of MO, only one peak of transformation (P_1_) was noted. The observed transformation was in a temperature range of −40 to −10 °C and had an endothermic character. This transformation was related to the melting of solidified mustard oil at the initial temperature. The peak at −21.4 °C was the melting point (T_m_) of this oil. Analysis of the DSC thermograms of bio-polyols containing boron and sulfur atoms in the temperature range of −40 to 180 °C showed the presence of three different types of transformations (P_1_–P_3_). The first type of transformation (P_1_) was associated with the glass transition of boron and sulfur-based bio-polyols. The peak of this transformation was a glass transition temperature (T_g_) of bio-polyol. Melting of bio-polyols was the second transformation (P_2_) observed on the thermogram. The maximum peak of this transformation (T_m_) was the melting point of bio-polyol. The last type of transformation (P_3_) observed on thermograms was associated with the beginning of the thermal decomposition of bio-polyols containing boron and sulfur atoms. Therefore, the T_d_ temperature was the temperature at which the process of thermal degradation of these bio-polyols begins. During this process, the destruction of ether bonds between the fat molecule and TDE or degradation of the chain from TDE may occur [51,52]. The temperatures of observed transformations from the DSC thermograms are presented in Table 8.

The thermal properties of bio-polyols depend on their molecular weight and chemical structure. The presence of additional side chains, additional functional groups or heteroatoms (such as boron and sulfur atoms) affects the occurrence of individual transformations and the value of their characteristic temperatures. An increase in the *M_n_* of bio-polyols can cause an increase in the degree of crystallization. Then, the mobility of amorphous chains is limited by neighboring crystals. This leads to a decrease in the glass transition temperature and an increase in the melting point [52]. In each of the analyzed bio-polyols containing boron and sulfur atoms, the appearance of the glass transition temperature T_g_ was observed in the temperature range of −40 to 180 °C. It was not found in the white mustard oil (MO) thermogram. In the case of the glass transition temperatures of the MP2 and MP3 bio-polyols, it can be concluded that they were at the same level and amounted to −30.3 and −32.1 °C, respectively. The T_g_ of the MP1 bio-polyol was much higher and was 18.3 °C. The difference in the values of glass transition temperatures resulted mainly from the difference in the chemical structure of new compounds. In the case of the MP2 and MP3 bio-polyols, the chemical structure was similar, with the difference that unsubstituted secondary hydroxyl groups were present in the MP3 bio-polyol (Scheme 7). In turn, a boric acid molecule was incorporated into each hydroxyl group in the MP1 bio-polyol. This molecule contained two unsubstituted OH groups that were capable of forming hydrogen bonds. This resulted in an increase in T_g_ by almost 50 °C in comparison with the MP2 and MP3 bio-polyols. A similar dependence was also noted for the temperatures of the second transformation (P_2_) associated with melting. The Tm of the MP2 and MP3 bio-polyols were at a similar level and were 14.9 and 11.2 °C, respectively. Hence, they were liquids of high viscosity at room temperature (Table 2). On the other hand, the melting point of the MP1 bio-polyol was much higher and was 75.9 °C, due to its chemical structure. Therefore, this bio-polyol was solid at room temperature. Analysis of the DSC results showed a difference in the intensity of P_2_ transformation peaks in MP1, MP2 and MP3. This difference resulted also from the different chemical structures of synthesized bio-polyols. The more interactions between the bio-polyol molecules (e.g., hydrogen bonds), the more heat is needed to effect this transformation [52]. The P_3_ transformation was associated with the thermal degradation of bio-polyols containing boron and sulfur atoms. It was observed only in the thermograms of the MP1 and MP2 bio-polyols. In the case of the MP3 bio-polyol, no transformation related to thermal decomposition was noted, similar to the MO thermogram. The transition occurring in bio-polyols above T_d_ is often associated with their temperature degradation. Macromolecules are broken up into small-molecule products [53]. The temperature T_d_ was significantly different for MP1 and MP2. It was 150.2 °C for the first bio-polyol and 75.9 °C for the second. The intensity of the peaks of this transformation were also different. This meant that the MP2 bio-polyol was much less thermally resistant than the MP1 or MP3 polyols. Starting thermal decomposition at a temperature of 75.9 °C may mean that this bio-polyol may not be suitable for the synthesis of polyurethane foams [54]. The presence of bonds such as an ether bond in the molecule favors thermo-oxidative degradation of these compounds at high temperatures [55]. In bio-polyols containing sulfur and boron atoms, such bonds occurred. They are formed as a result of the opening of the epoxide ring and the incorporation of the TDE molecules into the chains of fatty acids. The decomposition of these bonds could be the reason for the occurrence of the last (P_3_) transformation in bio-polyols.

### 3.5. Flammability Tests of Rigid Polyurethane/Polyisocyanurate Foams Modified by Bio-Polyols Containing Boron and Sulfur Atoms

Basic and extended tests were carried out as part of the research on the effect of new bio-polyols containing boron and sulfur atoms on the flammability of rigid polyurethane/polyisocyanurate foams. Basic research includes simple laboratory tests that give a specific parameter regarding the flammability of the material. This applies to the vertical and horizontal burning test, as well as the determination of a limiting oxygen index. The extended tests included the determination of a number of fire and smoke parameters. Such tests include cone calorimeter testing.

#### 3.5.1. Basic Flammability Tests

**Figure 5:**
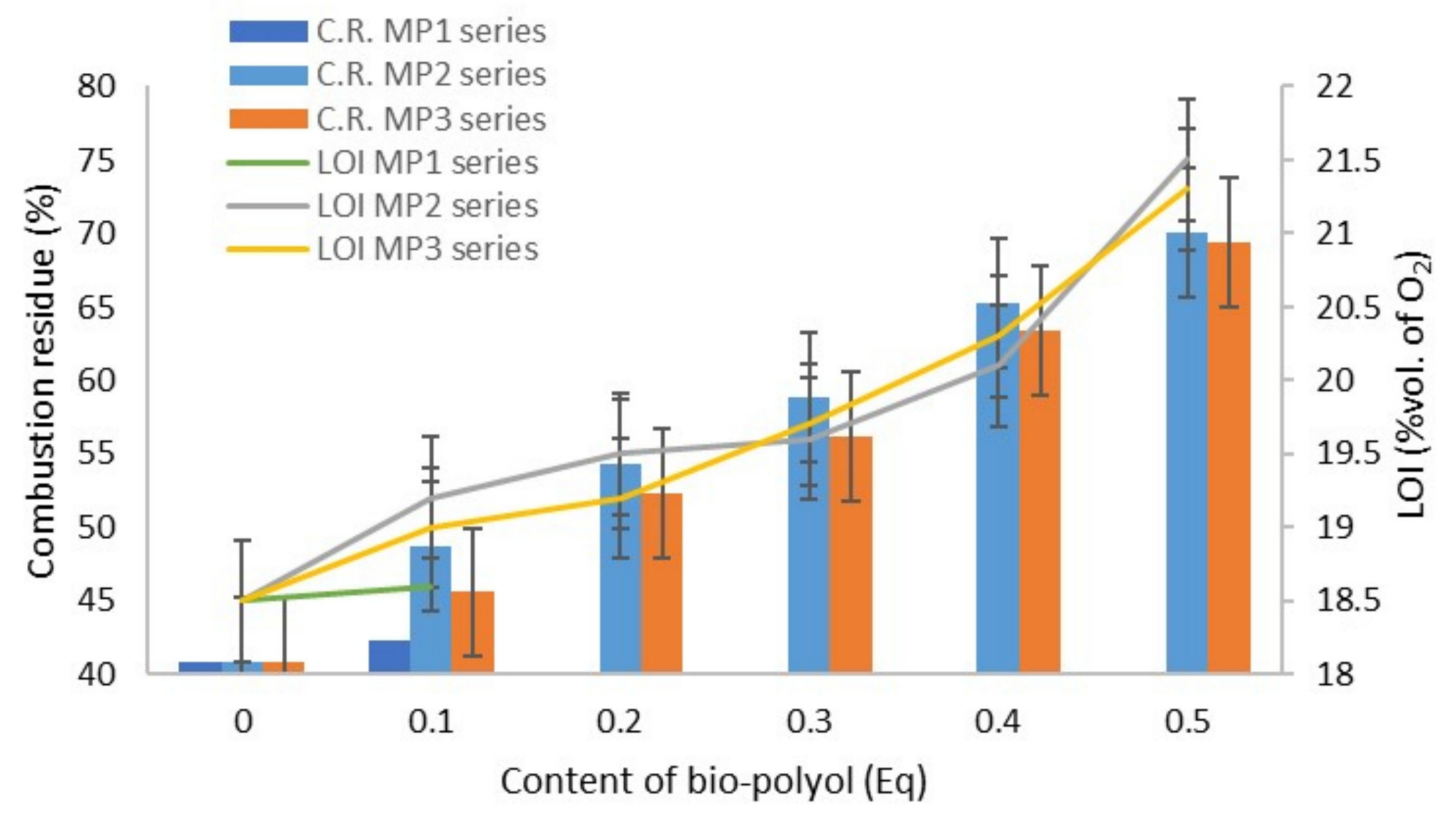
Dependence of combustion residues (C.R.) and LOI on the content of bio-polyols.

The basic flammability tests of the obtained rigid polyurethane/polyisocyanurate foams were carried out by three methods: vertical combustion test, horizontal combustion test and limiting oxygen index (LOI) test. All three flammability test methods make it possible to compare the flammability of foams after the addition of an internal flame retardant into the bio-polyol (e.g., flame-retardant elements like boron and sulfur atoms). The obtained combustion residues and LOIs of foams modified by bio-polyols are shown in Figure 5.

The flammability results from the vertical burning test method showed that with each test, the combustion residue increased. This was particularly evident in the case of the MP2 and MP3 series foams, in which up to half of the petrochemical polyol was replaced with bio-polyols. The reference foam, based on petrochemical polyol and without flame retardant, had a combustion residue of 40.9%. Replacing half of the petrochemical polyol with the equivalent amounts of the MP2 and MP3 bio-polyols increased this parameter to 70%. Additionally, in the case of the limiting oxygen index, a significant increase in this parameter was observed from 18.5 vol.% of O_2_ to more than 21 vol.% of O_2_. Crossing the 21 vol.% of O_2_ changes the classification of the material flammability from flammable to flame-retardant material. This is due to the fact that, in order to sustain combustion in the atmosphere, the amount of oxygen would have to be higher than its average amount in the air. In the case of the MP1 bio-polyol, a slight increase in the combustion residue from 40.9% to 42.3% and an increase in LOI value by 0.1% vol. of O_2_ was noted. It can be assumed that its content did not significantly improve the flammability of the material based on this bio-polyol. The situation could have been different if it had been possible to obtain a foam with a higher content of this bio-polyol. However, exceeding the content of 0.1 Eq of this bio-polyol made it impossible to obtain a rigid polyurethane/polyisocyanurate foam. The reason for the reduction of flammability of polyurethane foam materials based on bio-polyols was the presence of boron and sulfur heteroatoms. Based on the data obtained from elemental analysis (Table 4), it is known that the MP2 bio-polyol contained 7.44% of sulfur and 1.82% of boron, while the MP3 bio-polyol contained 5.49% of sulfur and 0.80% of boron. This amount was already sufficient to actively influence the combustion mechanism of the obtained foams. Based on the literature analysis, it is known that sulfur compounds are active in the gas phase, quenching oxidizing radicals during combustion. Boron acts in the condensed phase, creating a glassy layer on the surface of the material, cutting off the access of the oxidizing atmosphere to the material, which is fuel maintaining the combustion process [27,56]. The presence of both elements next to each other in the material has a synergistic effect because it works simultaneously in the gas and condensed phases. Consequently, the flame-retardant effect is higher than the sum of the effects of the individual elements [27].

The behavior of the rigid polyurethane/polyisocyanurate foams after the removal of the source of flame was observed in the horizontal combustion test. Obtained results showed that in each case the foams modified by bio-polyols containing sulfur and boron atoms did not burn after the removal of the fire source. According to ISO 3582:2002/A1:2008, these materials can be classified as self-extinguishing [57]. The reason for this was the decomposition of bio-polyols containing sulfur and boron atoms with the simultaneous formation of a glassy layer (from boron atoms) on their surface and the emission of volatile products with sulfur atoms that inhibit chemical reactions in a flame [33,55].

#### 3.5.2. Cone Calorimeter Test

The results of tests of fire and smoke parameters obtained after the flammability test with the cone calorimeter method are presented in Table 9.

The analysis of the research results obtained during the flammability test using the cone calorimeter method showed that in each case, an increase in the time to ignition (TTI) was noted after the use of bio-polyols containing sulfur and boron heteroatoms. It was 3 s for the reference foam, while it was extended for foams modified with the highest amount of bio-polyols to 4 s for MP1.1, 8 s for MP2.5 and 7 s for MP3.5, respectively. This meant that the increase in the content of bio-polyols had a positive effect on the flame retardancy of the foams because when the heat source was exposed to the sample, the surface of the foam decomposed with the release of gaseous sulfur compounds, which were not ignited by the igniter of the apparatus. In the cone calorimeter method, volatile compounds that are released from the sample during the exposition of the heat source from the cone have the greatest impact on the flammability of the material. The sample during the measurement was treated with a heat source of 50 kW and a temperature of 779 °C. If non-flammable compounds are released from the sample, the ignition of the sample is extended, or it does not occur at all. Very important parameters during a fire are the total amount of heat released and its rate. The more heat is generated during a fire, the greater the fire hazard the material presents. Additionally, if the heat is released very rapidly, it means that the material is extremely flammable and poses a very high risk [58]. In the case of foams based on bio-polyols containing sulfur and boron atoms, significant differences were noted in the total heat release (THR) and its release rate (HRR). For the reference foam, the THR was 24.3 MJ/m^2^ and the HRR was 278.90 kW/m^2^. After using the largest amounts of bio-polyols, both parameters significantly decreased to 23.6 MJ/m^2^ and 138.94 kW/m^2^ for MP1.1 foam, 19.5 MJ/m^2^ and 83.79 kW/m^2^ for MP2.5 foam and 19.7 MJ/m^2^ and 86.90 kW/m^2^ for MP3.5 foam. This meant that in each analyzed case the presence of sulfur and boron atoms reduced the total heat emission and inhibited its rate. The reason for this action was the formation of a glassy layer which not only blocked the access of the oxidizing atmosphere to the inside of the sample but also stopped the emission of heat to the outside. The parameters related to smoke emission during combustion are also very important apart from the fire parameters. In the event of a fire, the greatest risk is the smoke emitted during the combustion of various materials. Smoke is responsible for around 85% of fire-related deaths [59]. Therefore, it is very important not only to reduce the flammability of materials but also their smoke-emitting parameters. In the case of using bio-polyols containing boron and sulfur atoms, a decrease in the total smoke released (TSR) was noted in each case. A total of 912.4 m^2^/m^2^ of smoke was emitted from the reference foam during the test. The use of bio-polyols in the maximum amount in the formulation resulted in the reduction of this parameter to 752.5 m^2^/m^2^ for MP1.1, to 426.3 m^2^/m^2^ for MP2.5 and to 436.3 m^2^/m^2^ for MP3.5. In the case of the use of MP2 and MP3 bio-polyols, a more than two-fold reduction in smoke emissions was noted. Such action is very beneficial and confirms that the direction of research related to the introduction of heteroatoms into the foam is correct in terms of improving fire safety. The composition of the emitted smoke is also important. The amount of emitted carbon monoxide and carbon dioxide was also measured using the cone calorimeter method. For the reference foam, the amounts of CO and CO_2_ were 0.82 kg/kg and 6.2 kg/kg, respectively. The introduction of bio-polyols significantly reduced the emission of both gases to 0.0437 kg/kg and 1.32 kg/kg for the MP1.1 foam, 0.0163 kg/kg and 1.05 kg/kg for the MP2.5 foam and 0.0186 kg/kg and 1.09 kg/kg for the MP3.5 foam. All the obtained results of the flammability of rigid polyurethane/polyisocyanurate foams modified with bio-polyols containing sulfur and boron atoms allowed for the classification of these materials into the group of flame-retardant materials.

## 4. Conclusions

Three different bio-polyols based on mustard oil containing boron and sulfur heteroatoms were obtained as part of the research. Each bio-polyol was obtained by a different method aimed at introducing as many of the above-mentioned atoms as possible. Sulfur atoms were incorporated into the structure of bio-polyols with 2,2’-thiodiethanol, while boron atoms with boric acid and its esters with 2,2’-thiodiethanol. Obtained bio-polyols were subjected to detailed physicochemical, analytical and spectroscopic tests. It was noted that the synthesis method had a significant influence on the properties of the obtained bio-polyols. The greatest differences were noted for the bio-polyol obtained as a result of the esterification of boric acid with intermediate bio-polyol with incorporated 2,2’-thiodiethanol chains. The use of various synthesis methods also influenced basic parameters such as hydroxyl value, acid value, functionality, boron content and sulfur content. MP1 bio-polyol was characterized by the lowest hydroxyl value (55.25 mg KOH/g). In turn, in the case of MP2 and MP3 bio-polyols, the hydroxyl values were 188.34 and 178.42 mg KOH/g, respectively. A very important parameter of the obtained bio-polyols was the content of sulfur and boron. The highest boron content in the molecule was noted for the MP1 bio-polyol; it was 4.05%. For the others, the content of this element was 1.81% for MP2 and 0.80% for MP3. On the other hand, the highest sulfur content was noted for the MP2 bio-polyol (7.44%). In turn, for the other polyols, it was 5.08% for MP1 and 5.49% for MP3. The synthesized bio-polyols were used to obtain rigid polyurethane/polyisocyanurate foams. The obtained materials were subjected to four different flammability tests in order to determine the impact of boron and sulfur atoms on the flammability of these materials. All flammability tests showed that the use of bio-polyols significantly reduces the flammability of rigid polyurethane/polyisocyanurate foams. Using maximum amounts of MP2 and MP3 bio-polyols led to an increase in the combustion residue from 40.9% to 70%. The LOI also increased from 18.5% vol. of O_2_ to more than 21% vol. of O_2_. Tests with the use of a cone calorimeter showed that with each test, the fire and smoke parameters were significantly decreased. The research results carried out indicate the possibility of using the synthesized compounds for the production of polyurethane materials. The specific chemical structure and physicochemical properties of the synthesized bio-polyols indicate that they can be used as alternative substitutes to petrochemical raw materials used in the polyurethane industry.

## 5. Patents

Methods of synthesis of bio-polyols based on white mustard (*Sinapis alba*) oil contain-ing boron and sulfur atoms have been submitted for patenting—Polish Patent Application No. P.439000 “Method of synthesis of bio-polyol raw material containing sulfur and boron heteroatoms”.

## Figures and Tables

**Table 1:** Formulations of rigid polyurethane/polyisocyanurate foams with bio-polyols containing sulfur and boron atoms.

Foam Symbol	Rokopol RF551 (*Eq*) (g)	Bio-Polyol (*Eq*) (g)	Tegostab 8460 (g)	33% DABCO (g)	33% Potassium Acetate (g)	Distilled Water (g)	Purocyn B (*Eq*) (g)
**Ref.**	*1.0 * 66.79	*0.0 * 0.00	5.40	3.17	7.93	3.17	*3.7 * 250.60
**MP1.01**	*0.9 * 60.11	*0.1 * 50.77	6.15	3.62	9.04	3.17	*3.7 * 250.60
**MP2.01**	*0.9 * 60.11	*0.1 * 14.89	5.54	3.26	8.14	3.17	*3.7 * 250.60
**MP2.02**	*0.8 * 53.43	*0.2* *29.79*	5.67	3.34	8.35	3.17	*3.7 * 250.60
**MP2.03**	*0.7 * 46.75	*0.3* *44.68*	5.81	3.42	8.55	3.17	*3.7 * 250.60
**MP2.04**	*0.6 * 40.07	*0.4* *59.57*	5.95	3.50	8.76	3.17	*3.7 * 250.60
**MP2.05**	*0.5 * 33.39	*0.5* *74.47*	6.09	3.58	8.96	3.17	*3.7 * 250.60
**MP3.01**	*0.9 * 60.11	*0.1 * 15.72	5.54	3.26	8.16	3.17	*3.7 * 250.60
**MP3.02**	*0.8 * 53.43	*0.2 * 31.44	5.70	3.35	8.38	3.17	*3.7 * 250.60
**MP3.03**	*0.7 * 46.75	*0.3 * 47.16	5.86	3.45	8.61	3.17	*3.7 * 250.60
**MP3.04**	*0.6 * 40.07	*0.4 * 62.89	6.01	3.54	8.84	3.17	*3.7 * 250.60
**MP3.05**	*0.5 * 33.39	*0.5 * 78.61	6.16	3.63	9.07	3.17	*3.7 * 250.60

**Table 2:** Physicochemical properties of bio-polyols based on mustard oil.

Parameter	MP1	MP2	MP3
State of matter (at 20 °C)	solid	liquid	liquid
Color	brown	light-orange	orange
Smell	odorless	slightly sulfuric	slightly sulfuric
Density (g/cm^3^)	1.19 ± 0.01	1.09 ± 0.02	1.10 ± 0.01
Viscosity at 20 °C (mPa·s)	-	9400 ± 200	8800 ± 250
pH (-)	4.5 ± 0.2	7.0 ± 0.1	7.1 ± 0.1

**Table 3:** Analytical test results of bio-polyols based on mustard oil.

Parameter	MP1	MP2	MP3
Hydroxyl value (mg KOH/g)	55.25 ± 0.43	188.34 ± 2.91	178.42 ± 4.34
Acid value (mg KOH/g)	55.25 ± 0.43	2.42 ± 0.22	1.13 ± 0.13
Water content (%wt.)	0.10 ± 0.01	0.05 ± 0.01	0.05 ± 0.01

**Table 4:** Results of elemental analysis of bio-polyols based on mustard oil.

Element	MP1	MP2	MP3
Carbon (%)	54.53	65.20	66.20
Hydrogen (%)	10.16	11.48	11.69
Nitrogen (%)	0.00	0.00	0.00
Sulfur (%)	5.08	7.44	5.49
Oxygen (%)	26.18	14.06	15.82
Boron (%)	4.05	1.82	0.80

**Table 5:** Results of GPC analysis and functionality of bio-polyols based on mustard oil.

Parameter	MP1	MP2	MP3
M_w_ (g/mol)	4131	3998	2887
*M_n_* (g/mol)	1265	2654	2321
D (-)	3.27	1.51	1.24
f (-)	1.24	8.90	7.38

**Table 6:** Analysis of ^1^H NMR spectra of bio-polyols.

Number of Peak	Chemical Shift (ppm)	Type of Protons	Structure *
1	0.86–0.88	protons of ending groups –CH_3_	C**H**_3_
2	1.25–1.40	protons of CH_2_ groups in the fatty acid chain	C**H**_2_
3	1.60	protons of the β-CH_2_ group to the carbonyl (ester) group	–C**H**_2_–CH_2_–CO–
4	2.10–2.25	protons of α-CH_2_ groups to the olefin group	–C**H**_2_–CH=CH–
5	2.29–2.32	protons of the α-CH_2_ group to the carbonyl (ester) group	–C**H**_2_–CO–
6	2.70–2.80	protons of the α-CH_2_ group to the sulfide (thioester) group	–C**H**_2_–S–
7	3.52–3.60	protons of α-CH_2_ groups to the hydroxyl group	–C**H**_2_–OH
8	3.70–3.80	protons of hydroxyl groups at the end of the chain	–O**H**
9	3.85–4.10	protons of α-CH_2_ groups to the borate group	–C**H**_2_–OB<
10	4.15–4.28	methylene protons of glyceryl	–C**H**_2_–CH–CH_2_–
11	5.25	methane protons of glyceryl	C**H**_2_–CH–CH_2_–
12	7.27	protons of the hydroxyl group in boric acid	–B–O**H**

* The corresponding proton is marked in bold.

**Table 7:** Analysis of ^13^C NMR spectra of bio-polyols.

Number of Peak	Chemical Shift (ppm)	Type of Carbon	Structure *
1	14.30	carbons of ending groups	–**C**H_3_
2	22.70	carbons of penultimate groups	–**C**H_2_–CH_3_
3	27.20–29.80	carbons of CH_2_ groups in the fatty acid chains	–**C**H_2_–
4	31.90	carbons of α-CH_2_ groups to the carbonyl group	–CH_2_–OOC–**C**H_2_–
5	33.00–35.00	carbons of α-CH_2_ groups to sulfide group	–S–**C**H_2_–
6	61.10	carbons of β-CH_2_ groups to sulfide group	–S–CH_2_–**C**H_2_–
7	62.10	methylene carbons of glyceryl	–**C**H_2_–CH–**C**H_2_–
8	68.90	methane carbons of glyceryl	–CH_2_–**C**H–CH_2_–
9	73.86	carbons of α-CH groups linked to a hydroxyl group within the chain	>**C**H–OH
10	172.80–173.25	carbons of carbonyl groups	>**C**=O

* The corresponding carbon is marked in bold.

**Table 8:** Temperatures of characteristic transformations of MO and bio-polyols containing sulfur and boron atoms.

Sample	T_g_ (°C)	T_m_ (°C)	T_d_ (°C)
**MO**	-	−21.3 ± 0.4	-
**MP1**	18.3 ± 0.3	70.2 ± 0.7	150.2 ± 0.6
**MP2**	−30.3 ± 0.5	14.9 ± 0.5	75.9 ± 0.6
**MP3**	−32.1 ± 0.4	11.2 ± 0.3	-

**Table 9:** Results of cone calorimeter test.

Parameter	TTI	THR	MLR	HRR	t_HRRmax_	Released CO	Released CO_2_	TSR
Unit	(s)	(MJ/m^2^)	(g/m^2^·s)	(kW/m^2^)	(s)	(kg/kg)	(kg/kg)	(m^2^/m^2^)
**Ref.**	3	24.3	10.22	278.90	10	0.8201	6.20	912.4
**MP1.01**	4	23.6	8.34	138.94	10	0.0437	1.32	752.5
**MP2.01**	5	21.6	6.38	107.34	20	0.0322	1.22	542.6
**MP2.02**	5	21.3	6.02	102.86	30	0.0274	1.11	499.4
**MP2.03**	7	21.1	5.42	98.02	40	0.0226	1.07	473.8
**MP2.04**	7	20.3	5.21	92.76	45	0.0178	1.06	439.7
**MP2.05**	8	19.5	5.11	83.79	45	0.0163	1.05	426.3
**MP3.01**	4	22.0	6.79	115.33	20	0.0321	1.27	535.7
**MP3.02**	5	21.7	6.19	108.36	35	0.0246	1.18	493.3
**MP3.03**	6	21.4	5.55	104.32	40	0.0216	1.11	482.0
**MP3.04**	7	20.5	5.39	93.76	40	0.0190	1.10	447.3
**MP3.05**	7	19.7	5.35	86.90	43	0.0186	1.09	436.3

## Data Availability

Data is contained within the article.

## Supplementary Materials

The following supporting information can be downloaded at: www.mdpi.com/1996-1944/16/9/3401/s1, Table S1: Apparent density of obtained foams.
