# Supplementary material for: Comparing the Properties of Bio-Polyols Based on White Mustard (Sinapis alba) Oil Containing Boron and Sulfur Atoms Obtained by Various Methods and Checking Their Influence on the Flammability of Rigid Polyurethane/Polyisocyanurate Foams
Source: Materials (Basel). 2023 Apr 26;16(9):3401. doi: 10.3390/ma16093401 (PMC10180400; doi:10.3390/ma16093401)
Supplement: Supplementary file 1 [file materials-16-03401-s001.zip › materials-2291539-supplementary.pdf]

materials

Comparison of the properties of bio-polyols based on white mustard (*Sinapis alba*) oil containing boron and sulfur atoms obtained by various methods and checking their influence on the flammability of rigid polyurethane/polyisocyanurate foams

Marcin Borowicz[1,*], Marek Isbrandt[1], Joanna Paciorek-Sadowska[1] and Paweł Sander[1]

[1] Department of Chemistry and Technology of Polyurethanes, Faculty of Materials Engineering, Kazimierz Wielki University, J. K. Chodkiewicza Street 30, 85-064 Bydgoszcz, Poland
* Correspondence: m.borowicz@ukw.edu.pl (M.B.); Tel.: +48-52-34-19-286

1. Apparent density of foams modified by new bio-polyols

Table S1. Apparent density of obtained foams.

Foam symbol	Apparent density (kg/m^3)
**Ref.**	51.48 ± 1.32
MP1.01	52.93 ± 2.14
MP2.01	51.02 ± 1.74
MP2.02	50.12 ± 1.05
MP2.03	47.39 ± 2.21
MP2.04	45.89 ± 1.14
MP2.05	45.03 ± 1.37
MP3.01	49.99 ± 0.89
MP3.02	48.02 ± 1.14
MP3.03	47.78 ± 1.43
MP3.04	46.83 ± 1.75
MP3.05	46.13 ± 1.54
